# Research Projects How They Sprout, Bloom, and Wither Away

**DOI:** 10.1002/tcr.202400153

**Published:** 2025-04-16

**Authors:** Reinhard W. Hoffmann

**Affiliations:** ^1^ Fachbereich Chemie der Philipps Universität Marburg Hans Meerwein Str. 4 D-35032 Marburg Germany

**Keywords:** History of Chemistry, Reflections on Research

## Abstract

The conception, initiation, major achievements, and termination of twelve different research projects in our research group are described. Covering a period of four decades, they mirror the changing trends and focusses of organic chemistry in the second half of the last century.

## Graphical abstract

What is most exciting in doing research? Not to reach the stated goal, but the unforseen encounters on the way, and to realize their implications!

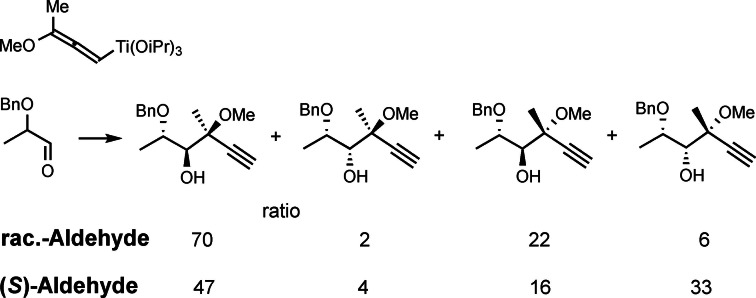

## 1 Introduction

**Figure 1:**
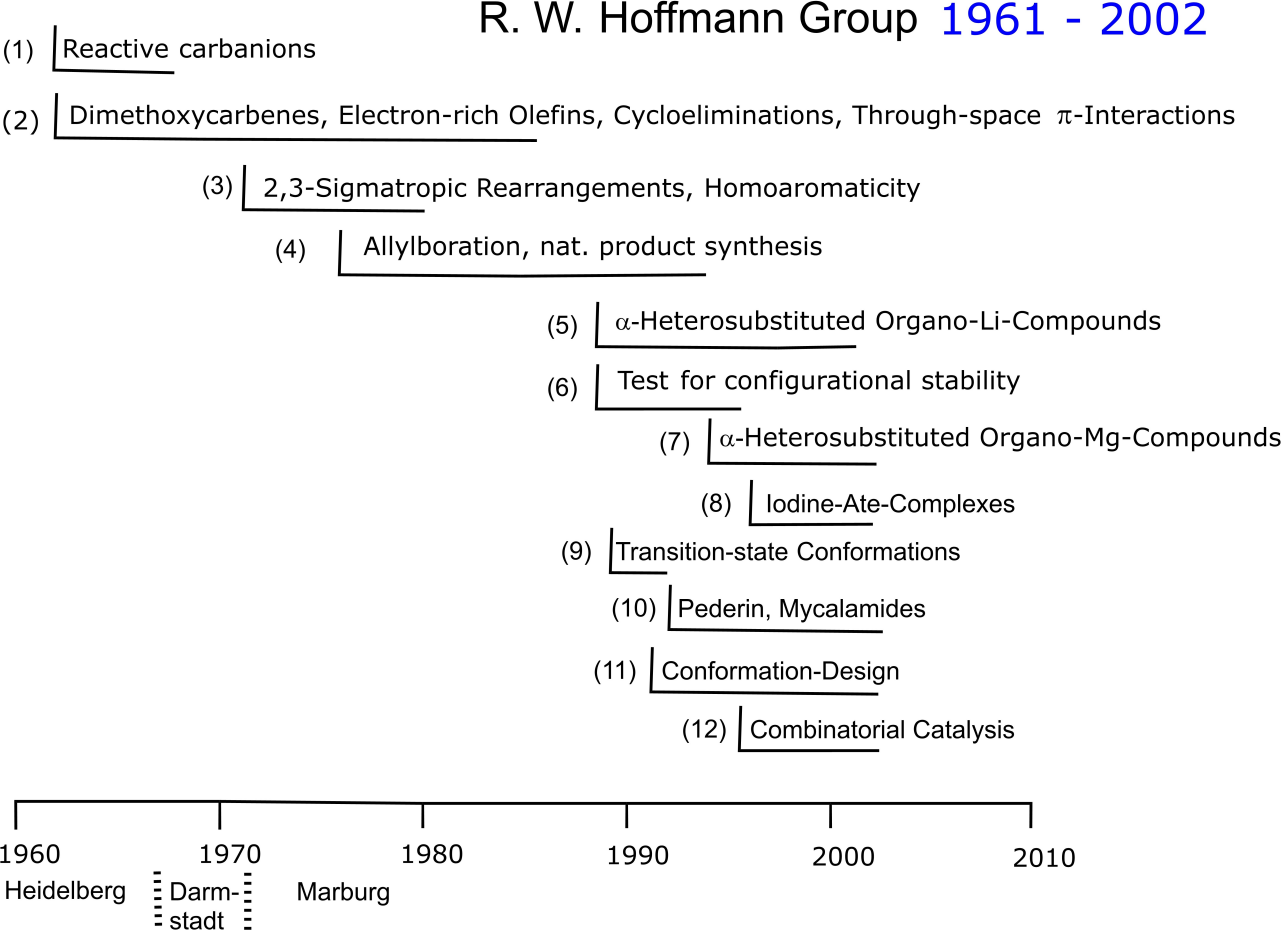
Projects (section number) and their timelines covered in the following essay.

Looking back at ones scientific oeuvre, one sees at first the achievements, the successes and failures. However, the route to these hardly ever followed a straight course. Some projects had a long and varied life, others ended soon in a dead end. Some projects were loosely connected with others, some in turn arose seemingly unexpectedly. It are these germinations of projects, leading hopefully to an extended bloom period before withering away, that describe the course of an extended voyage through Organic Chemistry. Given these vagaries, I tried in the following essay to present twelve projects or series of related projects of our research group covering a forty‐year period. An overview is given in Figure 1.

In the individual sections, the projects will not be covered comprehensively. Rather their conception, benchmarks, and factors that led to a modification of the project will be addressed. The resulting compilations illustrate the diversity of factors that influenced the development as well as success or failure of a project.

## 2 The Outset

Und Jedem Anfang Wohnt ein Zauber inne

H. Hesse, 1941

At the University of Heidelberg in 1961, when I got the chance to start independent research in the group of Georg Wittig, physical organic chemistry was beginning to grow roots in Germany. The younger generation of scientists was fascinated by reaction mechanisms, especially reactive intermediates.

**Scheme 1:**
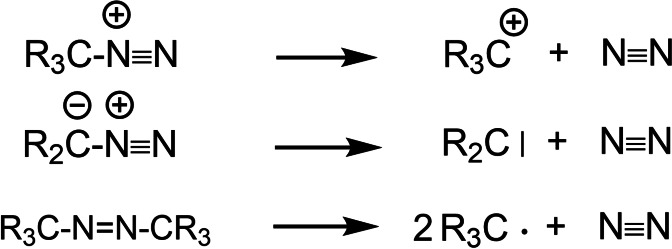
Reactive intermediates by deazotation.

Reactive intermediates, such as carbenium ions, carbenes, or free radicals are energy‐rich species. In order to study them under mild conditions, their generation was frequently coupled with the formation of a low‐energy coproduct, such as dinitrogen (Scheme 1).

**Scheme 2:**
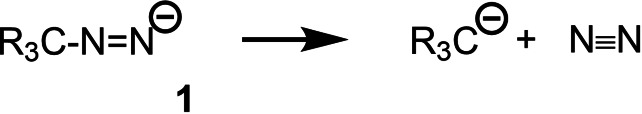
Deazotation to generate carbanions.

Wittig's group followed the encompassing goal to study carbanion‐chemistry as a counterpart to the already established carbenium‐ion chemistry of Hans Meerwein. In this regard, it was surprising that there were no studies of carbanions as short‐lived intermediates. Accordingly, I found it appealing to expand the above methods to generate short‐lived reactive carbanions (Scheme 2):

I envisioned to generate carbanions in protic media and to characterize such reactions, vic. rearrangements of carbanions, that are able to outcompete protonation in protic media.

**Scheme 3:**
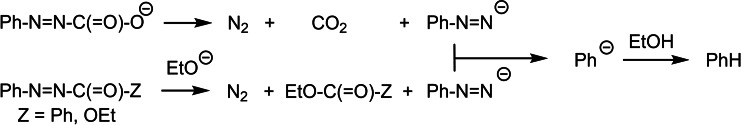
Generation of phenyl‐diazenyl anions and their deazotation.

The diazenyl‐anions **1** can, however, not be handled as such, and are reactive intermediates themselves. They have to be generated as nucleofugic entities from suitable precursors. The known base‐induced heterolytic fragmentation of aryl‐azo‐carbonyl compounds[^1^, ^2^, ^3^, ^4^] can be interpreted to provide an entry‐point to the studies we intended (Scheme 3).

**Scheme 4:**
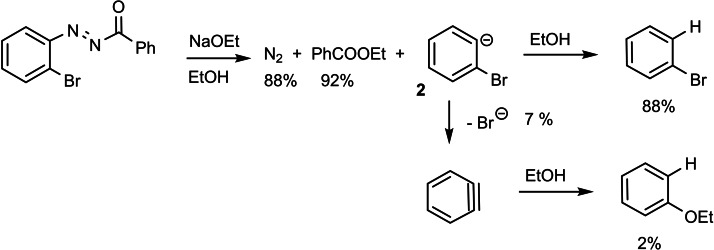
Generation of 2‐bromo‐phenyl anion.

On this basis, I chose the following test reaction as a means to generate the 2‐bromophenyl anion in protic media. This test worked out right at the first attempt^[5]^ and provided not only bromobenzene, but also a small amount of phenetole, hopefully arising from loss of bromide from the 2‐bromophenyl anion **2** and subsequent addition of ethanol to dehydrobenzene (Scheme 4). The name “dehydrobenzene” had been given to this intermediate by G. Wittig, who first postulated its formation. Later the term “benzyne” became accepted.

**Scheme 5:**
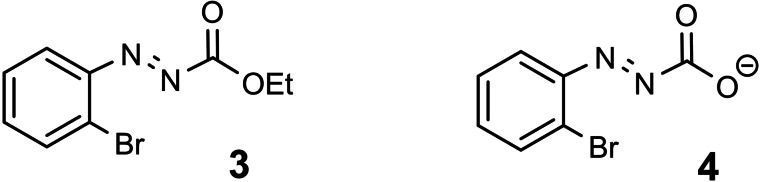
Related precursors to the 2‐bromo‐phenyl anion.

Similar results were obtained on fragmentation of the azo compounds **3** and **4** indicating the generality of this route to the 2‐bromophenyl anion **2**.[^5^, ^6^] (Scheme 5)

**Scheme 6:**
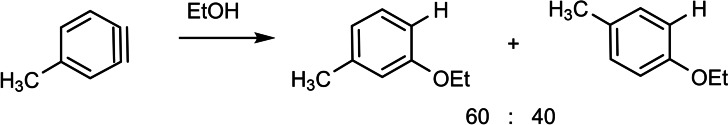
Proof of aryne formation.

The intermediacy of dehydrobenzene in these reactions was, in addition, secured by placement of a methyl‐substituent, leading to the meta‐ and para‐isomers in a known characteristic ratio.[^5^, ^6^] (Scheme 6)

**Scheme 7:**
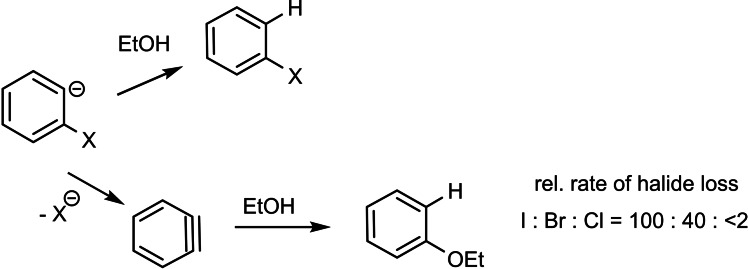
Relative rates of halide loss from 2‐halo‐phenyl anions by reference to their protonation rate as internal standard.

Using this technique of heterolytic fragmentation of arylazocarbonyl compounds, the rates of halide loss from the 2‐halo‐phenyl anions could be estimated relative to the rate of protonation of these anions:^[7]^ (Scheme 7)

**Scheme 8:**
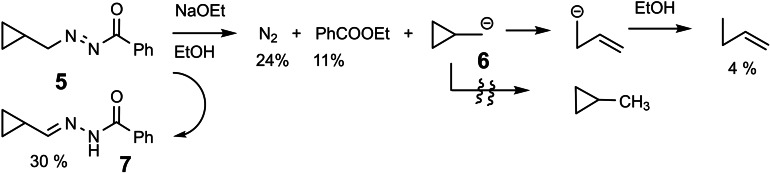
Attempts to generate cyclopropylcarbinyl anions.

The success in generating *aryl*‐anions could unfortunately not be carried over to the generation of *alkyl* anions. This became apparent, when we set out to study the ring‐opening of the cyclopropylcarbinyl anion **6** to the butenyl anion. Upon attempted fragmentation of the azo compound **5** the hoped for fragmentation turned out to be only a minor reaction path, whereas isomerization of **5** to the more stable hydrazone **7** became the main process.^[8]^ (Scheme 8)

**Scheme 9:**
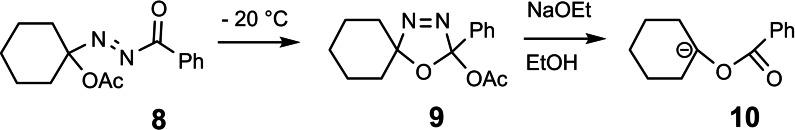
Attempts to generate α‐acyloxy carbanions.

The results obtained are quite typical for what one can call planned research. Challenges arise when the unexpected comes to the surface: E.g. compound **8** should not be amenable to an undesired isomerization to a hydrazone. It was synthesized in the hope of generating a hitherto unaddressed α‐acyloxyalkyl anion. However, compound **8** unexpectedly rearranged already at low temperatures to the Δ^3^–1,3,4‐oxadiazoline **9**.^[9]^ (Scheme 9) This class of compounds later opened up a rich scientific harvest in the hands of J.Warkentin.^[10]^ That means that we missed to recognize its true potential at this point.

But, compound **9** was nevertheless of interest in the context of our planned study. Upon treatment with sodium ethoxide, it underwent likewise a fragmentation to liberate the sought after α‐acyloxyalkyl anion **10**. The latter was, however, rapidly protonated suppressing any hoped for anionic acyl‐shift to provide an α‐keto‐alkoxide.^[9]^

## 3 Cycloelimination of Carbenes from Bicyclo[2 : 2 : 1]heptadienes

**Scheme 10:**
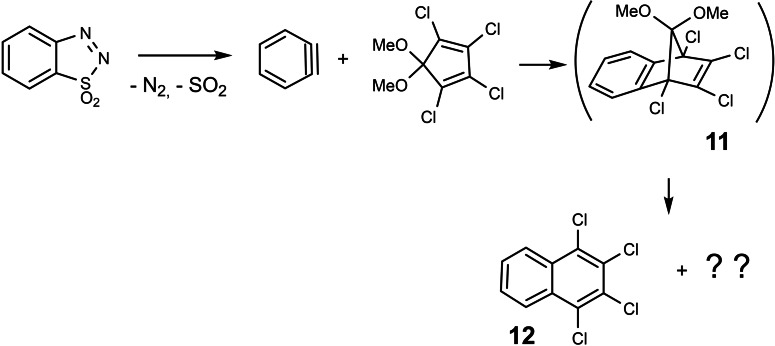
Attempted Diels‐Alder‐addition of dehydrobenzene.

During my stay as a postdoc in Wittig's group, I intended to trap benzyne by tetrachloro‐5,5‐dimethoxy‐cyclopentadiene in a Diels‐Alder addition, expecting to obtain the adduct **11**. (Scheme 10)

However, rather than compound **11**, I isolated in unspectacular yield 1,2,3,4‐tetrachloro‐naphthalene **12**. In hindsight, I may have chosen inappropriate conditions on reaction‐workup, because it was shown later, that compound **11** may well be prepared by trapping benzyne with tetrachloro‐5,5‐dimethoxy‐cyclopentadiene.^[11]^ At any rate, our experiment was not published and fell into oblivion, until when my first graduate student asked for a research project. I remembered the findings above and wanted to use this chance for starting a second research line.

**Scheme 11:**
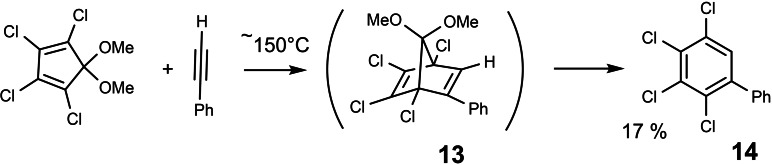
Thermal loss of CH_3_O−C−OCH_3_ bridge from a bicyclo[2 : 2 : 1]heptadiene.

En route from the expected compound **11** to the product **12**, the bridge CH_3_O−C−OCH_3_ had been lost from the skeleton. Could it be that dimethoxy‐carbene can be generated by thermolysis of bicyclo[2 : 2 : 1]heptadiene derivatives? A literature search revealed a further example of a thermal loss of a CH_3_O−C−OCH_3_ bridge.[^12^, ^13^] (Scheme 11)

**Scheme 12:**
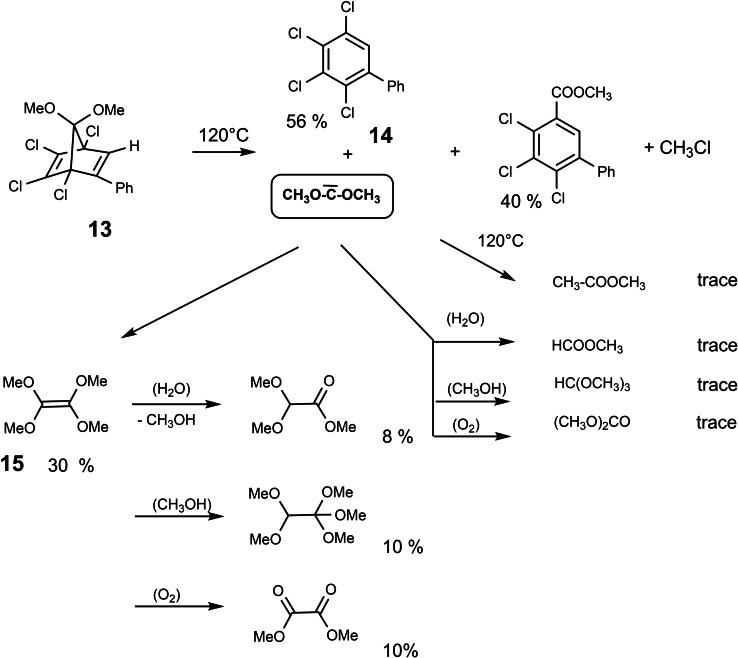
Reaction paths in the thermolysis of the bicyclo[2 : 2 : 1]heptadiene **13**.

Again, the fate of the bridge in **13** had not been investigated. In the end, I succeeded to convince Helmut Häuser, my fist graduate student, to tackle this project. He began by preparing compound **13** via a milder indirect route, and studied its thermal decomposition in the 120 to 140 °C temperature range. The aromatic products, mainly compound **14**, were readily characterized. Whereas on dealing with the volatile products we made all sorts of beginners mistakes (dissolving the products in CCl_4_ for the sake of unproblematic NMR‐spectra, not being aware of the fact that the main product **15** reacts with CCl_4_). However, eventually we got a clear picture of the reaction scheme:^[14]^ (Scheme 12)

**Scheme 13:**
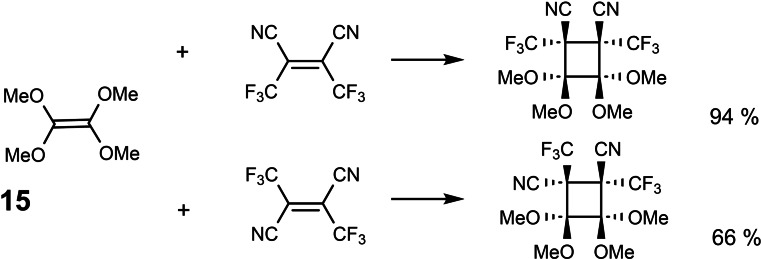
Stereospecific polar [2+2]‐cycloadditions.

The obtained products indicated indeed the intermediacy of dimethoxy‐carbene. The individual reactions shown in the scheme could be reproduced by separate experiments. The main volatile product of the pyrolysis of **13** was tetramethoxyethylene **15**, the dimerization product of dimethoxy‐carbene. Its yield could be increased to about 60 %. The pyrolysis of **13** proved then to be the key to a flow of changing research activities of our group described in the following:

Initially we studied the chemistry of tetramethoxyethylene **15** as a prototypical electron rich alkene, i. e. one with a high‐lying HOMO.^[15]^ We thus found a spectacular case of a stereospecific [2+2]‐cycloaddition: (Scheme 13)

**Scheme 14:**
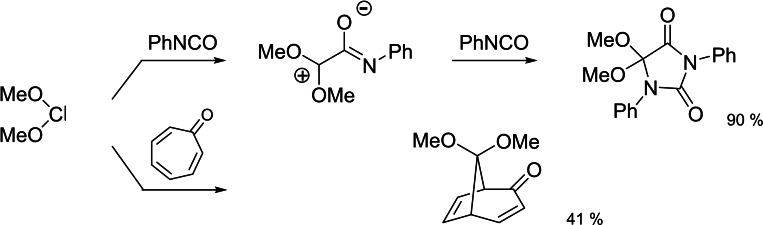
Reactions of dimethoxycarbene with electrophilic π‐systems.

Subsequently we studied the behavior of the moderately nucleophilic dimethoxy‐carbene generated by the thermolysis of **13**.[^16^, ^17^] (Scheme 14)

**Scheme 15:**
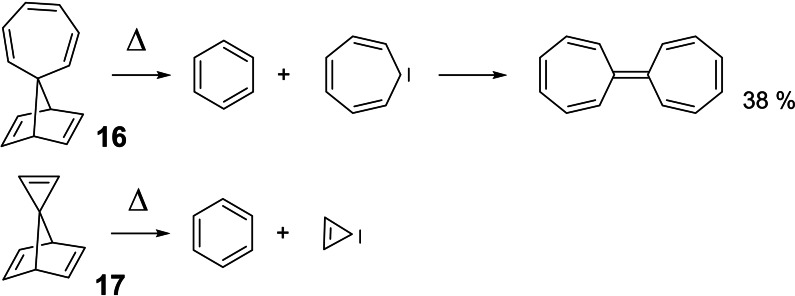
Generation of other nucleophilic carbenes from bicyclo[2 : 2 : 1]heptadienes.

Once the obligatory studies for dealing with a new compound had been completed, we went one step back to clarify, which bridges in a bicyclo[2 : 2 : 1]heptadiene system may be eliminated as a carbene. In this endeavor we found the thermolyses of compounds **16** and **17**,[^18^, ^19^] which allowed the first characterization of cyclopropenylidene in a low temperature matrix. (Scheme 15)

**Scheme 16:**
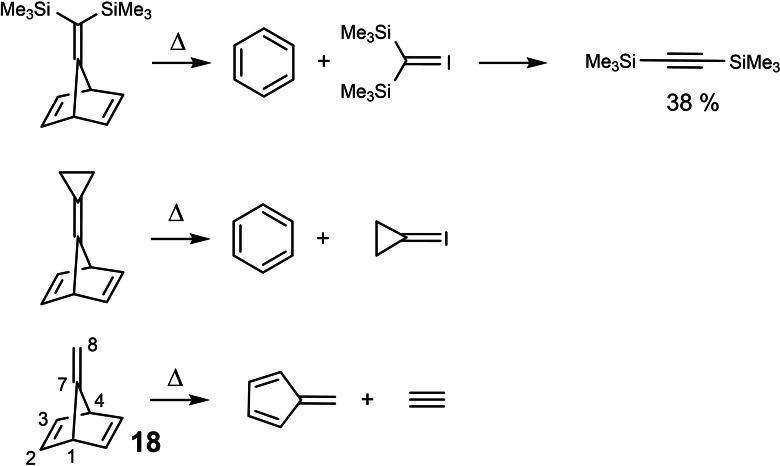
Thermolysis of 7‐alkylidene‐norbornadienes.

Vinylidenes too could be generated by thermolysis of appropriate bicyclo[2 : 2 : 1]heptadienes.^[20]^ But as thermolysis of compound **18** showed, this held not for all alkylidene‐norbornadienes. (Scheme 16)

We recognized that a carbene‐cycloelimination will be preferred over a retro‐Diels‐Alder cleavage, when the cycloelimination generates a *nucleophilic* carbene, that is, when an electronic interaction of the substituents at C‐7 (or C‐8) with the C‐1−C‐7−C‐4 bond system destabilizes the latter. In a more general view, these considerations allowed a deeper insight into the factors that determine selectivity on thermal bond‐cleavage.^[21]^

**Scheme 17:**
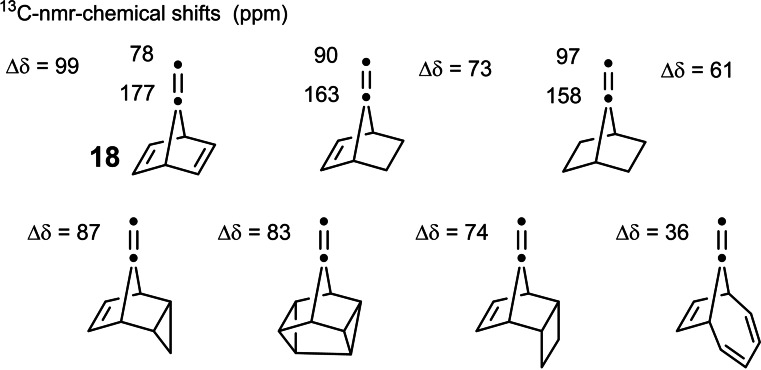
Polarization of the semicyclic double bond in 7‐alkylidene‐norbornadienes.

But the journey wasn't yet over at this point: We noted that compound **18** showed unusual ^13^C‐NMR‐signal positions that indicated a substantial polarization of the C‐7, C‐8‐double bond.^[22]^ (Scheme 17) The polarization was attributed to a through space molecular orbital interaction between the π‐systems, which was manifest from the photoelectron spectra. This led us to study this kind of molecular orbital interactions as a genuine subject. These orbital interactions caused characteristic differences in the reactivity of the semicyclic double bond towards electrophiles,[^23^, ^24^] which are not covered here.

**Scheme 18:**
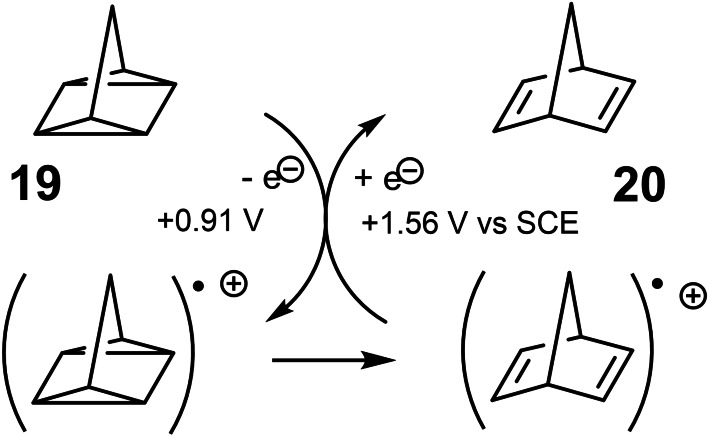
Electron‐transfer catalyzed isomerization of quadricyclanes.

When considering orbital interactions between occupied molecular orbitals, photoelectron spectroscopy turned out to be handy. From the photoelectron spectra it was evident that quadricyclane **19** is easier to ionize than norbornadiene **20**.^[25]^ This has a bearing on the isomerization of quadricyclane‐derivatives to the corresponding norbornadienes, a reaction we frequently used to prepare the required 7‐substituted norbornadienes (by application of transition‐metal catalysts). (Scheme 18) When quadricyclane **19** is oxidized to its radical cation, the latter could isomerize to the more stable radical cation of norbornadiene.^[26]^ In due course, this is a stronger oxidant than the radical cation of **19** and hence could oxidize neutral **19**,^[25]^ being itself reduced to neutral norbornadiene **20**. This defines a pathway for an electron‐transfer catalyzed isomerization of **19** to **20**, a process that would merely require an electrode held at a potential between that of the oxidation potential of **19** and **20**! While we did not study this option in detail, we treated **19** with a 0.01 equivalents of a triarylaminium radical cation salt as oxidant, and indeed recorded a quantitative conversion of **19** into norbornadiene **20**.^[27]^

**Scheme 19:**
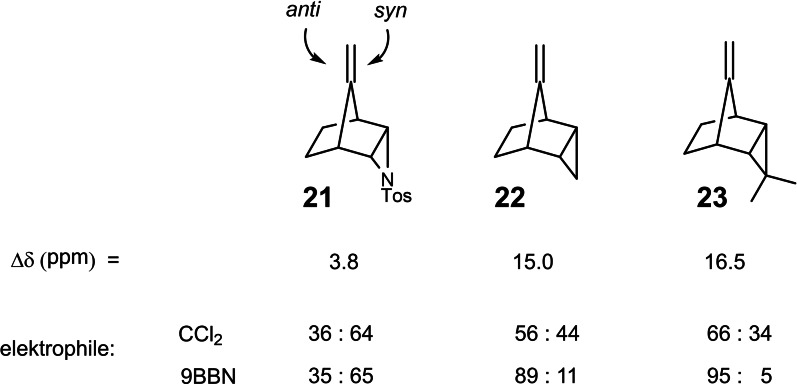
One‐sided electronic interactions of Walsh‐orbitals with a semicyclic double bond.

Eventually there was still an aftermath to our engagement with the bicyclo[2 : 2 : 1]heptadienes. In 1975, as in the previous years, I sought occasions to discuss chemistry with some major players of organic chemistry. I just went to visit them. So I left Marburg by car one morning and could reach the ETH‐ Zürich by noon. This allowed the afternoon for interaction with the Zürich organic chemists. On driving back, I reflected on the conversations. The one with Albert Eschenmoser concerned the electronic structure of enamines, cf. ref. [28]. The question was, whether the electron density of the C−C‐double bond could be different on the top and bottom face due to an electronic interaction with the lone electron pair on the nitrogen atom. This should become manifest in the facial selectivity of the double bond, an effect that could not be addressed separately from steric effects. On the way, back in the leisure of driving it occurred to me that we would have with the compounds **21** to **23** a nearly perfect system to address such a question. (Scheme 19)

The stereochemical situation of the semicyclic double bond is essentially constant in the compounds **21**–**23**.^[29]^ Whereas the extent of the orbital interaction between the semicyclic double bond and the Walsh orbitals of the cyclopropane ring differs, as seen from the Δδ values in the ^13^C‐nmr‐spectra. Any difference in the π‐electron density at the top and bottom face of the semicyclic double bond should then be reflected in the anti/syn selectivity on reaction of these compounds with representative electrophiles. As the above data suggest such a relationship could be substantiated at least in a qualitative manner.^[30]^

Taken together, a flow of discoveries related to bicyclo[2 : 2 : 1]heptadienes arose over more than twenty years from a single experiment in 1961 – seemingly done in an inappropriate manner. In hindsight, we could have not predicted which doors this experiment opened. An impressive testimony to the power of unplanned research.

## 4 Dead Ends

### 4.1 Stereochemistry of the Allylsulfoxide/Allylsulfenate Rearrangement

**Scheme 20:**
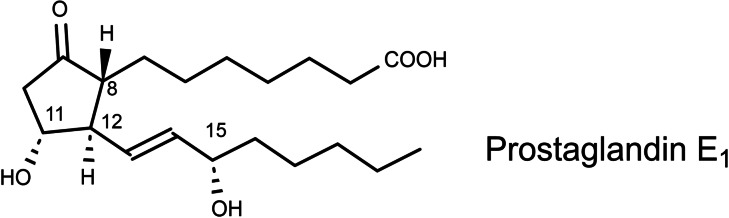
Structure of Prostaglandin E_1_.

In November 1968 I attended the 12^th^ R.A.Welch Foundation Conference on Chemical Research, Organic Chemistry. E.J. Corey disclosed during the conference his initial success in the synthesis of prostaglandins. This opened my eyes to the importance of the prostaglandins, and henceforth I joined the rapidly growing number of chemists, who engaged in conceiving prostaglandin‐syntheses.^[31]^ (Scheme 20)

**Scheme 21:**
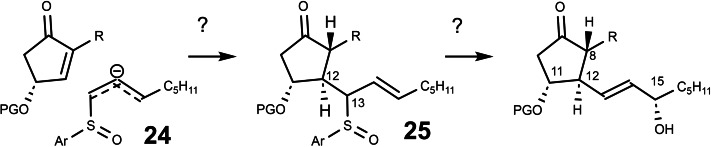
Connecting the stereocenter ar C‐15 with the other stereocenters?

In the years that followed, the number of publications on prostaglandins increased in an exponential fashion, pointing clearly to the main stereochemical problem of all attempted syntheses of prostaglandins: The stereogenic centers at C‐8, C‐11, and C‐12 are neighboring and can be generated by classical approaches of 1,2‐asymmetric induction. In contrast, the stereogenic center at C‐15 is spatially separated from the former and has to be generated in a separate effort. To remedy this situation, I wondered whether it would be possible to generate the stereogenic center at C‐15 alongside the ones at C‐8, C‐11, and C‐12? Could chirality transfer from a stereogenic center at C‐13 to C‐15 effect this? The stereogenic center at C‐13 would be a temporary one, which could be hopefully set by being neighboring to the one at C‐12. (Scheme 21) I envisaged a Mislow/Evans allylsulfoxide/allylsulfenate‐rearrengement[^32^, ^33^] for this purpose, leading to the following plan:

This plan held several imponderabilities, which had to be clarified in a first effort:

– Does a chirality transfer succeed originating from allylsulfoxides **25** which epimerize rapidly at C‐13?
– How does the extent and direction of chirality transfer depend on the configuration at C‐13 and the sulfoxide *sulfur* stereogenic centers?
– Do allylsulfoxide anions **24** enter into a Michael addition into α,ß‐unsaturated ketones?
– Do the anions **24** add via the α‐ or the γ‐C‐atom to α,ß‐unsaturated ketones?

**Scheme 22:**
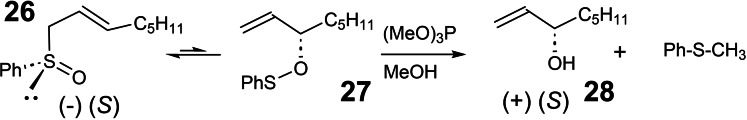
Chirality transfer in the Mislow/Evans rearrangement.

The first task was to find a viable access to enantiomerically pure allylsulfoxides such as **26**, considering that such allylsulfoxides racemize at temperatures above −10 °C. That done,[^34^, ^35^] we evaluated the extent and direction of the chirality transfer on Mislow/Evans rearrangement of relevant allylsulfoxides: Starting from enantiomerically pure allylsulfoxide **26**, the resulting octenol **28** was obtained with merely 29 % optical purity.^[35]^ (Scheme 22)

**Scheme 23:**
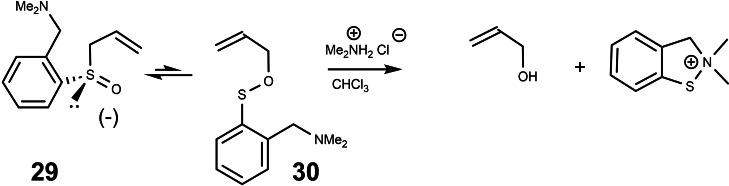
Intramolecular cleavage of an allylsulfenate intermediate.

One of the prerequisites for a high level of chirality transfer is, that the cleavage of the allylsulfenate **27** is more rapid than its return to the allylsulfoxide **26**. The cleavage of **27** by trimethyl phosphite is a bimolecular process, whereas its return to **26** is a monomolecular reaction. Therefore, unless trimethyl phosphite is applied in high excess, the return of **27** to **26** will dominate after a threshhold value of conversion. This situation could be remedied by rendering the cleavage of the allylsulfenate monomolecular as well=intramolecular. (Scheme 23) To this end, we devised the model system **29**:

For the sulfenate **30** to be cleaved, a proton is needed, i. e. acid has to be present. This acid though, should not protonate the tertiary amine, the effective thiophile, to a stoichiometric extent. Hence, we chose as acid a salt of a secondary amine. With this setup, the model reaction worked fine.^[36]^ How did we know, that the conversion of **29** to **30** is irreversible? The rate of product formation (measured by ^1^H‐nmr) was found to be identical to the rate by which the optical rotation of **29** disappeared! As much pleasure we had in devising a molecule **29** with a built‐in mechanism for self‐destruction, as little impact, though, this had on the scientific community.

**Figure 2:**
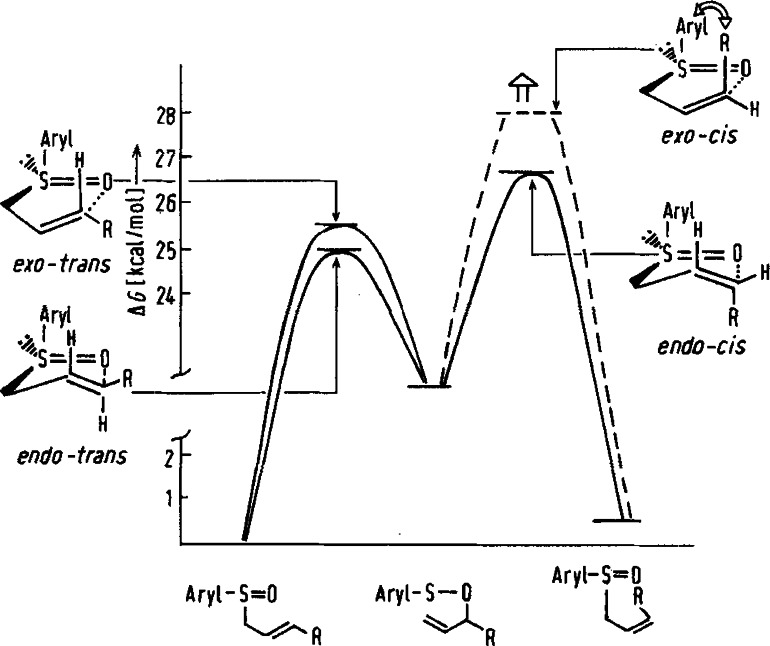
Energy diagram for the [2.3]sigmatropic rearrangements of allylsulfoxides (Reproduced from Angew.Chem.**1979**, 91, 625 with permission from John Wiley and Sons).

Once the reaction setup was such that the rearrangement of **26** to the allysulfenate **27** was irreversible, the low enantiomeric purity of the octenol obtained reflects the competition of two diastereomeric reaction pathways for the [2,3]sigmatropic rearrangement leading to (*S*)‐ and (*R*)‐octenol respectively, as can be seen from the following energy diagram. (Figure 2)

The study was then extended to the corresponding Z‐allyl‐sulfoxide. Here, a high level of chirality transfer could be realized as the competing *exo‐cis*‐ and *endo‐cis*‐transition states differ significantly in energy. With these results, a complete set of reaction pathways for a [2,3]sigmatropic rearrangement^[32]^ was characterized.

Coming back to the originally intended prostaglandin syntheses: In order to realize a high level of chirality transfer, the *cis*‐isomer corresponding to compound **25** would be required! Yet we saw no route to generate this in isomerically pure form.

Anyhow, we looked nevertheless briefly into the feasibility of the Michael‐additions of allylsulfoxide anions **24**. On addition of **24** to cyclopentenone, no satisfactory regioselectivity (α‐ vs. γ‐addition) could be realized, cf. also.^[37]^ At this point we saw no longer a chance to realize a prostaglandin synthesis along the lines outlined above. At least, the influence of the C‐13 stereogenic center on the direction and extent of the chirality transfer originating from compound **25** has been later clarified in a prostaglandin synthesis by G. Stork.^[38]^

The bottom line, enticed by the idea of realizing a stereoselective synthesis of prostaglandins we embarked on a study of the allyl‐sulfoxide/allylsulfenate rearrangement. While the prostaglandin synthesis could not even in parts be realized, we gained a comprehensive understanding of the allyl‐sulfoxide/allylsulfenate rearrangement and [2,3]sigmatropic rearrangements in general.^[39]^

### 4.2 Homoaromatic Anions

**Scheme 24:**
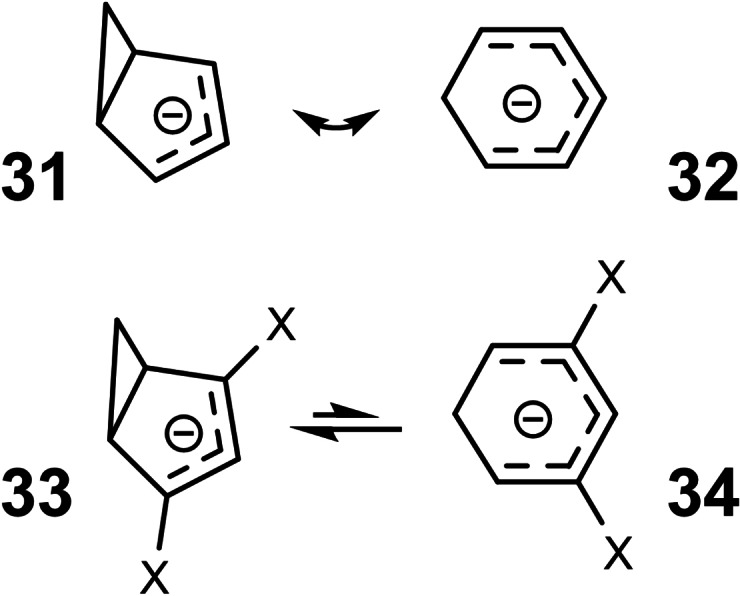
Potential homoaromatic delocalization in anionic π‐systems?

Around 1970 homoaromaticity was clearly a topic of interest in organic chemistry. Homoaromaticity was accepted in cationic species (vic. homotropylium ions). Regarding neutral compounds (norcaradiene/cycloheptatriene), it was controversial.^[40]^ Nothing was known concerning homoaromatic *anions*. A case of interest could be the system **31**/**32** (Scheme 24)

On the basis of valence‐bond considerations, bond delocalization had the highest chance, when the contributing structures were of approximately equal energy. This certainly did not hold for the system **31**/**32**, formally related to the norcaradiene/cycloheptatriene system, as **31** is destabilized by ca. 25 kcal/mole due to ring strain. It occurred to me that anion **31** could be selectively stabilized by anion‐stabilizing substituents, cf. **33**, the position of which would to a first degree not affect the stability of anion **34**. Hence, we wondered, which substituents X (NO_2_, CN, COOR, CHO) would stabilize anion **33** to an extent that it will be of similar energy to anion **34** in equilibrium. In more general terms, we wanted to deploy charge stabilization against ring strain, hoping that when **33** and **34** are of similar energy, the system could be tested for the presence of homoaromaticity.

**Scheme 25:**
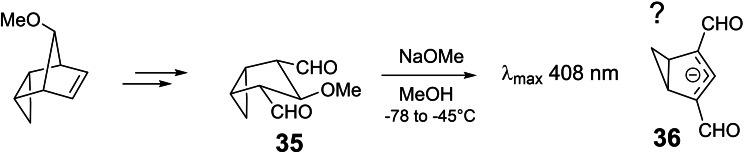
Synthesis of a potentially homoaromatic anionic π‐system.

We aimed at compound **33** in which X is an aldehyde group, as the latter could easily be converted to other anion‐stabilizing substituents. The access to suitable precursors turned out to be recalcitrant. Eventually, the following inroute was realized:^[41]^ (Scheme 25)

Yet, when compound **35** was treated with base, an expected based on model compounds, yet transient UV‐absoprtion at 408 nm was recorded. This could be an indication for the formation of anion **36**, which evaded further characterization by unspecified conversion to benzenoid species. Unfortunately, we lacked the equipment and expertise to characterize the transient intermediate. It is probably the CH_2_‐group in the anion **34** that triggers the undesired aromatization. Accordingly, the intended study would require analogs of compound **33** in which the CH_2_‐group is replaced by a CR_2_‐moiety. In view of the lengthy inroute, we refrained from embarking on such an extended study.

**Scheme 26:**
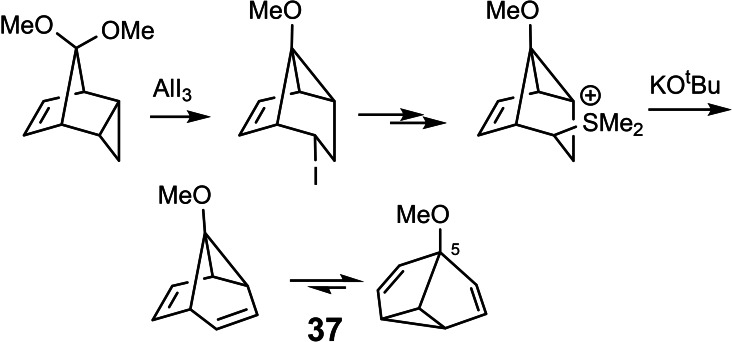
Route to 5‐methoxy‐semibullvalene.

Nevertheless, this study opened an unexpected entry into the arena of valence tautomeric compounds with fluxional structures: Starting from one of the intermediates in the synthesis of **35** we could realize an access to 5‐methoxy‐semibullbvalene **37**, wherein the methoxy‐group prefers a placement away from the cyclopropane ring.^[42]^ (Scheme 26)

Dead ends? Adversities and roadblocks are commonplace in any research project. Yet, research projects that run into a dead end may however be more valuable than those that led to the expected result! When an expected result is reached, nothing essential has been learned. The state of knowledge has been confirmed but not expanded. When adversities force a researcher to stop a project, deficiencies in our knowledge become obvious, pointing to where further research is needed. And, as in the examples above, the unanticipated windfall may be more valuable than the originally aspired goal.

**Scheme 27:**
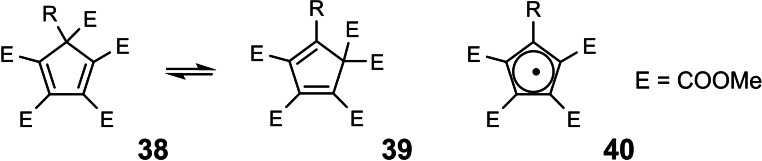
1,5‐sigmatropic ester shifts.

Concerning such unanticipated windfall: our study on substituent effects (of R) on the rate of 1,5‐sigmatropic ester shifts on the penta‐methoxycxarbonyl‐cyclopentadiene backbone **38** to **39**.^[43]^ (Scheme 27) This study provided a test of the theoretical analysis of sigmatropic rearrangements by Epiotis,^[44]^ whereby the rates of sigmatropic rearrangements could be modelled with reference to the SOMO‐energy of the corresponding backbone radical, cf. **40**.

**Scheme 28:**
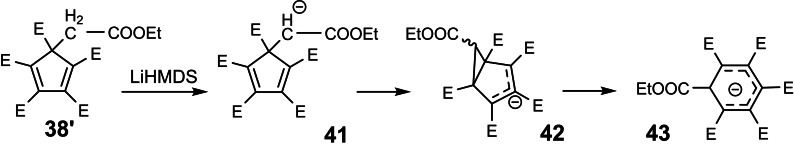
Synthetic transformations en route to the bicyclo[3 : 1:0]hexane skeleton.

But how did we come to initiate this study? A clue can be found in the following scheme in the paper^[45]^ dealing with follow up reactions of the starting materials **38**. (Scheme 28)

The conversion of **42** into **43** corresponds to the equilibration **31**, **32**, delineated above, and reveals that the sequence **41** → **42** was the original goal of the undertaking, during which the thermal sigmatropic ester shift was encountered, i. e. the windfall.

## 5 Allylboronates in Stereoselective Synthesis

After moving in 1970 from Darmstadt to Marburg, I spent my first sabbatical in summer 1975 at the University of Wisconsin, Madison, where I had been visiting professor before in 1968/69. The chemistry department there was highly interactive. The colleagues met when possible at 2 : 30 p.m. for a cup of coffee in a small social room; and this room had been assigned to me as a temporary office. Accordingly, I had ample opportunity to discuss current results or new ideas with Chuck Casey, Harlan Goering, Steve Nelsen, Hans Reich, Barry Trost, Ed Vedejs, Howard Whitlock, or Howard Zimmerman; certainly a paradisiacal situation for a scientist.

**Scheme 29:**
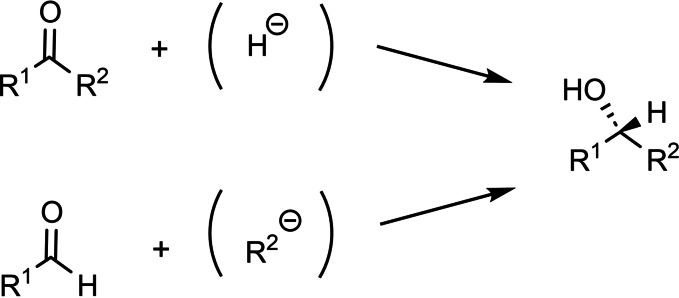
Prochirality of ketones vs. aldehydes.

In the 1970s the development of methods for enantioselective synthesis was a leading theme in Organic Chemistry. Of primary importance were methods to generate secondary alcohols in an enantioselective manner. To do this by reducing prochiral ketones with chiral reductants had the inherent disadvantage that the enantiotopic faces of a ketone are hard to differentiate when R^1^ and R^2^ are structurally similar. (Scheme 29)

**Scheme 30:**
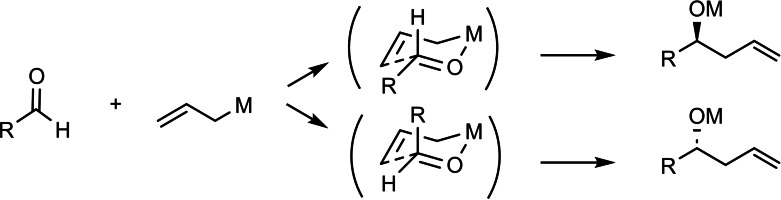
Allylmetallation of aldehydes.

The enantiotopic faces of a carbonyl group are, however, maximally differentiated on addition of an anionic residue to an aldehyde. An optimal differentiation would be expected for reactions that proceed via a compact, late transition state. The aspired differentiation is caused by differences in the interaction of the residues of the chiral reagent with R^1^ and H of the aldehyde substrate. Accentuated and defined interactions would be expected for addition reactions that proceed via cyclic transition states. These considerations led us to turn to the addition of allyl‐metal compounds to aldehydes: (Scheme 30)

**Scheme 31:**
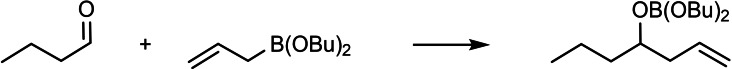
Allylboration of aldehydes.

To maximize steric interactions (and their differences!) in the transition state, the newly formed metal‐oxygen bond should be as short as possible. Moreover, the reaction should only be minimally exothermic in order to guarantee a late, and hence compact transition state. We reasoned that these conditions would be met by M=B(OR)_2_. Moreover, importantly, the use of boronic esters would provide an easy means to attach chiral information, in order to achieve the desired asymmetric induction. At this point a literature search showed that the basic reaction, the addition of allylboronates to aldehydes was known.^[46]^ (Scheme 31)

**Scheme 32:**
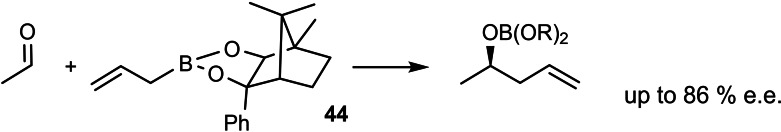
Enantioselective allylboration of aldehydes.

So, all was set to study the modification of this reaction by chiral glycols. After my return to Marburg the next graduate student, Thomas Herold, tested pinanediol and bornanediol as chiral auxiliaries and attained on addition of the allyboronates to acetaldehyde asymmetric inductions of 8, respectively 12 % e.e.. After this proof of principle, crude modelling of the competing transition states led us to prepare the allyboronate **44**, which reached up to 86 % e.e. on addition to acetaldehyde, at that time a highly respectable value.^[47]^ (Scheme 32)

**Scheme 33:**
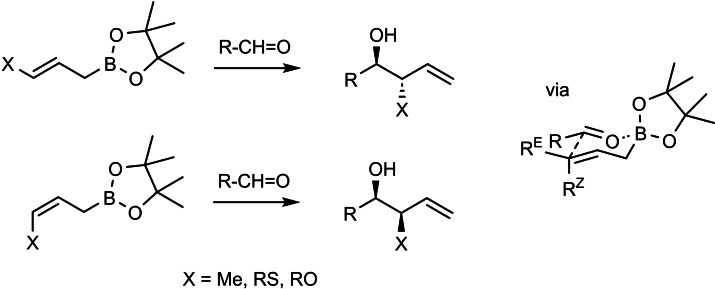
Simple diastereoslectivity on allylboration of aldehydes.

This then provided the starting point for a long success story: The addition of γ‐substituted allylboronates to aldehydes proceeded with close to perfect simple diastereoselectivity: (Scheme 33)

**Scheme 34:**
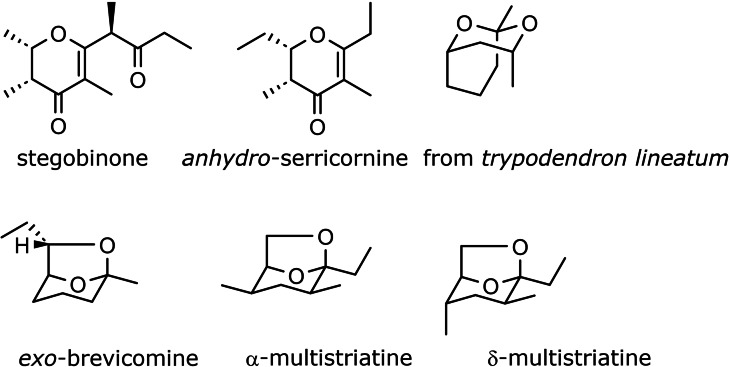
Natural products accessed by allylboration of aldehydes.

In combination with the chiral auxiliary, cf. **44**, these findings allowed us to prepare simple chiral natural products, the synthesis of which would seem trivial today. (Scheme 34) They were in those days, however, valid targets for stereoselective synthesis.[^48^, ^49^, ^50^, ^51^, ^52^]

**Scheme 35:**
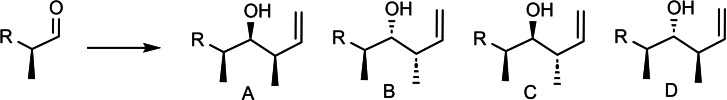
Stereocontrol needed in the synthesis of polyketide natural products.

In order to attain the synthesis of more complex natural products, it was necessary to realize at will the following transformations leading to each of the stereotriads A–D.^[53]^ (Scheme 35)

**Scheme 36:**
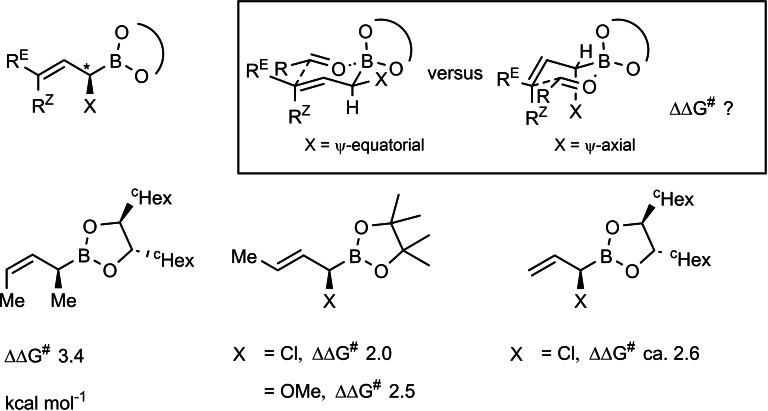
Chirality transfer on allylboration with α‐substituted allylboroantes.

The asymmetric induction originating from the chiral starting aldehyde had in some situations to be boosted, which could be effected with the chiral auxiliary, cf. **44**. But, when the asymmetric induction from the starting aldehyde had to be overruled, allylbroronates of the type **44** were insufficient. Hence, a second generation of α‐chiral allylboronates had to be developed, one, in which the asymmetric induction is based on a direct chirality transfer via the cyclic transition states.^[54]^ (Scheme 36)

**Scheme 37:**
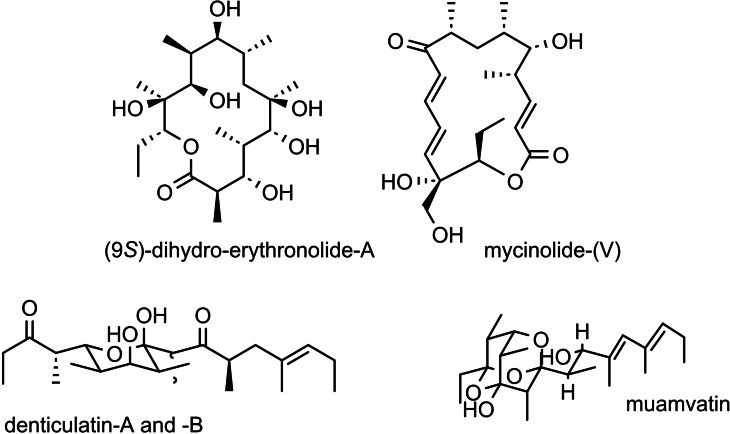
Polyketide natural products synthesized by means of second generation allylboronates.

The level of asymmetric induction by these reagents can be quantified by the ΔΔG^≠^ values, the energy difference between the competing diastereomeric transition states. With these second‐generation chiral allylboronates we then realized the synthesis of the following polyketide natural products, wherein the construction of the molecular skeleton by the asymmetric allylboration reactions was the key issue.[^55^, ^56^, ^57^] (Scheme 37) allylboronates. For further examples see section 11.

Our synthesis of (9*S*)‐dihydro‐erythronolide‐A^[58]^ is even 30 years later one of the shortest realized syntheses, a testimony to the efficiency that can be achieved by using the second generation chiral allylboronates. This development, though, could not at all be foreseen in 1975, when the enantioselective allylboration reaction was conceived.

## 6 α‐Heterosubstituted Alkyllithium Compounds

In 1987 a collaborative research project „Organometallic Compounds as Selective Reagents in Organic Synthesis“ was started in Marburg by the Deutsche Forschungsgemeinschaft. When coordinating this project, I realized that an expansion of the initial research program was in need.

**Scheme 38:**
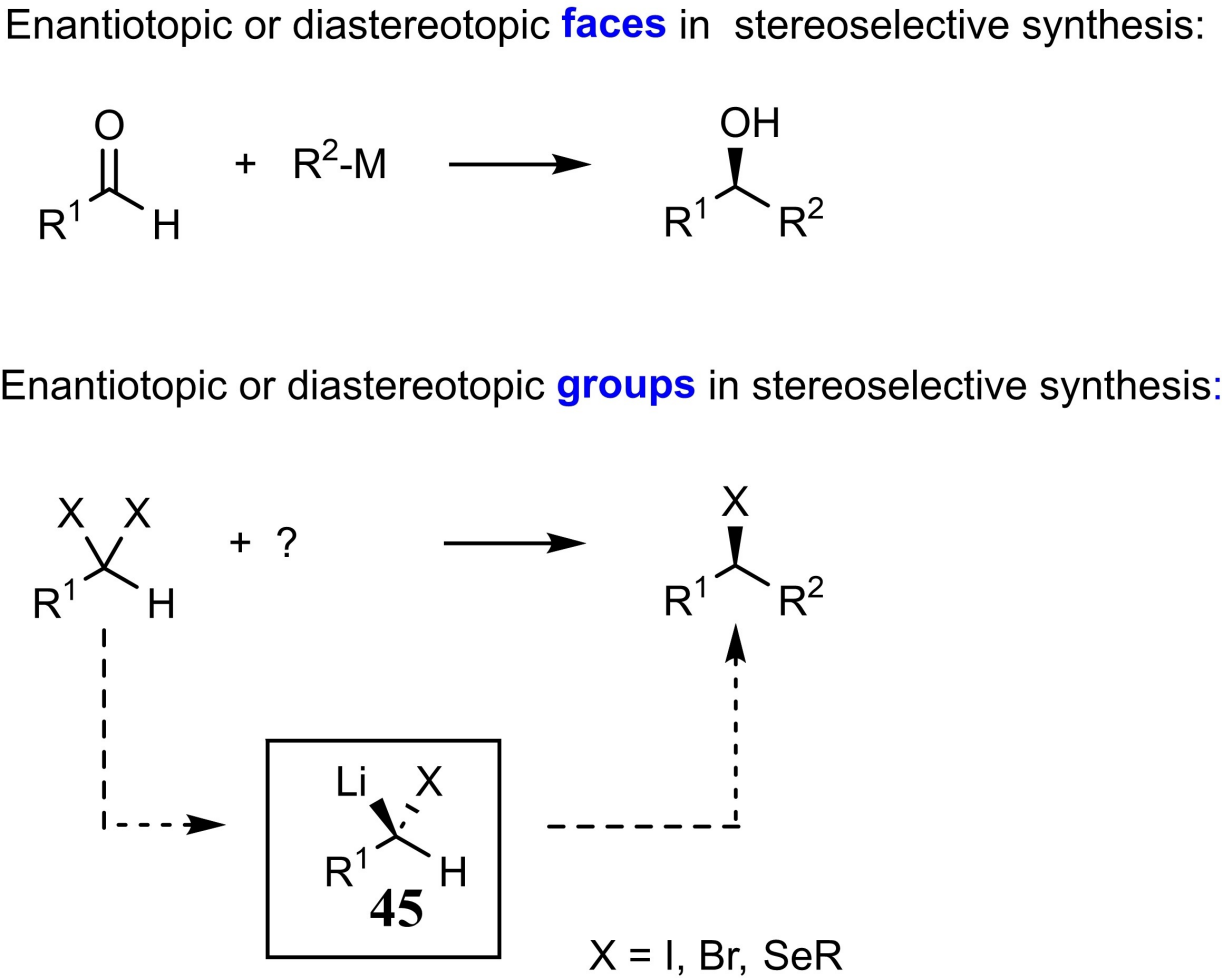
Heterotopic groups and faces in stereoselective synthesis.

This led me to think about the essence of stereoselective synthesis. Creation of a stereogenic center begins from a prochiral entity, usually a *planar* prochiral group (C=O, C=NR; C=CR_2_), whose faces are either enantio‐ or diastereotopic. In the stereogenic reaction, the heterotopic faces of the prochiral group have to be differentiated. Planar prochiral groups are, however, not the only representation of prochirality. As nmr‐spectra amply demonstrate, there are also molecules containing carbon atoms carrying enantiotopic or diastereotopic groups. This kind of prochirality had not found systematic scrutiny in stereoselective synthesis, while isolated examples had been reported. This line of thought therefore opened the chance to expand the arsenal of stereoselective synthesis in a fundamental manner. Hence, we set out within the context of the collaborative research project to clarify, whether chiral α‐heterosubstituted alkyllithium compounds **45** can be generated by differentiation of enantiotopic or diasterotopic groups. (Scheme 38)

So much regarding the de novo concept – when I lectured 1988 about the first results at Harvard university, E. J. Corey commented „I wish, I would have had this idea!“

**Scheme 39:**
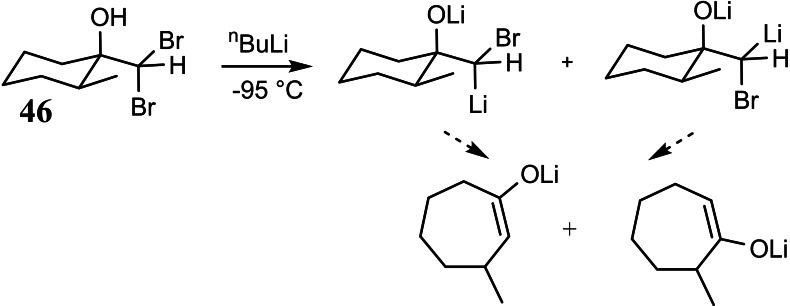
Bromine/lithium exchange of diasterotopic bromine atoms.

We turned to the bromine/lithium exchange reaction at *gem*‐dibromoalkanes as a means to generate carbenoids, a reaction that was pioneered by my former Heidelberg colleague Gert Köbrich, who had passed away much too early. This reaction had already been studied in breadth^[59]^ and provided even an example **46**, in which the bromine atoms involved were diastereotopic.^[60]^ There was no report, however on the diastereoslectivity in the bromine/lithium exchange on compound **46**. The investigation had been terminated just at a point, where stereochemical aspects would have expanded the picture.^[61]^ (Scheme 39)

**Scheme 40:**
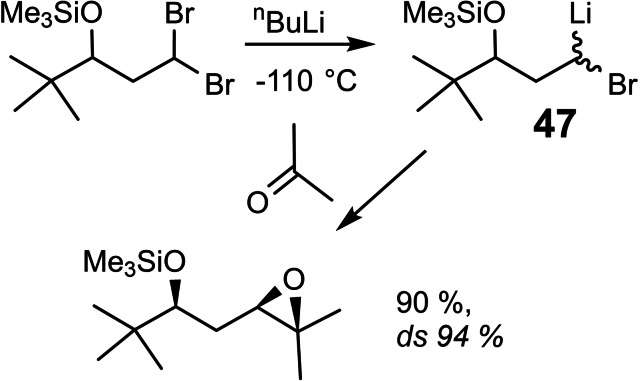
Bromine/lithium exchange of diasterotopic bromine atoms.

We found ourselves, thus in the unusual situation, that all necessary preliminary experiments had already been performed by other groups. Accordingly, Rainer Stürmer could land a full hit in his first week as an undergraduate research participant:^[62]^ (Scheme 40)

**Scheme 41:**
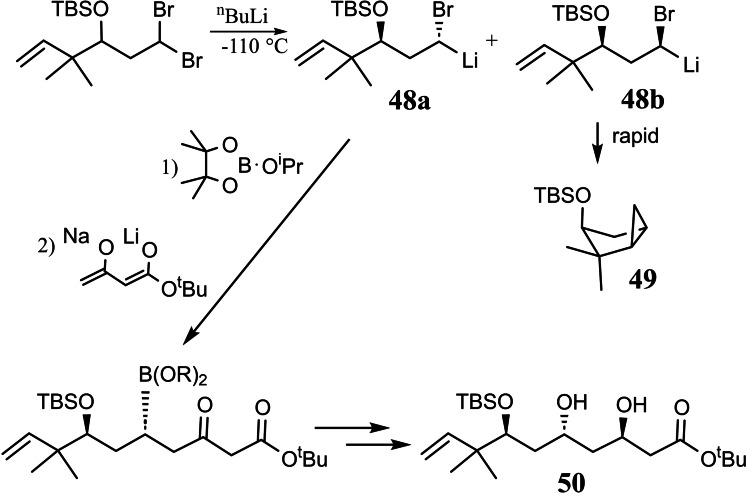
Bromine/lithium exchange of diasterotopic bromine atoms.

He succeeded to generate an α‐bromoalkyllithium compound **47** of defined configuration, and he established conditions, under which these carbenoids are configurationally stable. The potential of this finding is illustrated by the following reaction sequence, in which the major product‐diastereomer **48 a** has been carried on to a bryostatin building block **50**,^[63]^ (Scheme 41)

while the minor product‐diastereomer **48 b** cyclized rapidly to the bicyclo[3 : 1:0]hexane **49**.^[64]^ The latter process commands interest by itself, as it established for the first time the stereochemistry of a cyclopropanation reaction at the metal‐bearing carbon atom of a carbenoid.

**Scheme 42:**
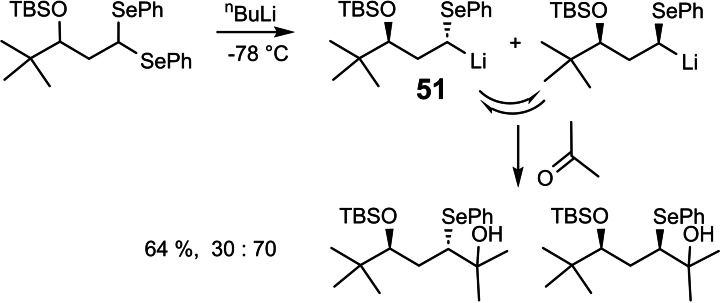
Selenium/lithium exchange of diasterotopic selenium atoms.

The investigations on the selective exchange of diastereotopic groups were then extended to selenoacetals.^[65]^ (Scheme 42)

A detailed study revealed that α‐seleno‐alkyllithium‐compounds **51** are not configurationally stable under the reaction conditions, hence, leading to Curtin‐Hammett control of product formation. But even though, stereoselective transformations may be realized, when the isomer ratio of the α‐seleno‐alkyllithium‐compounds **51** is biased by chiral ligands.^[66]^

**Scheme 43:**
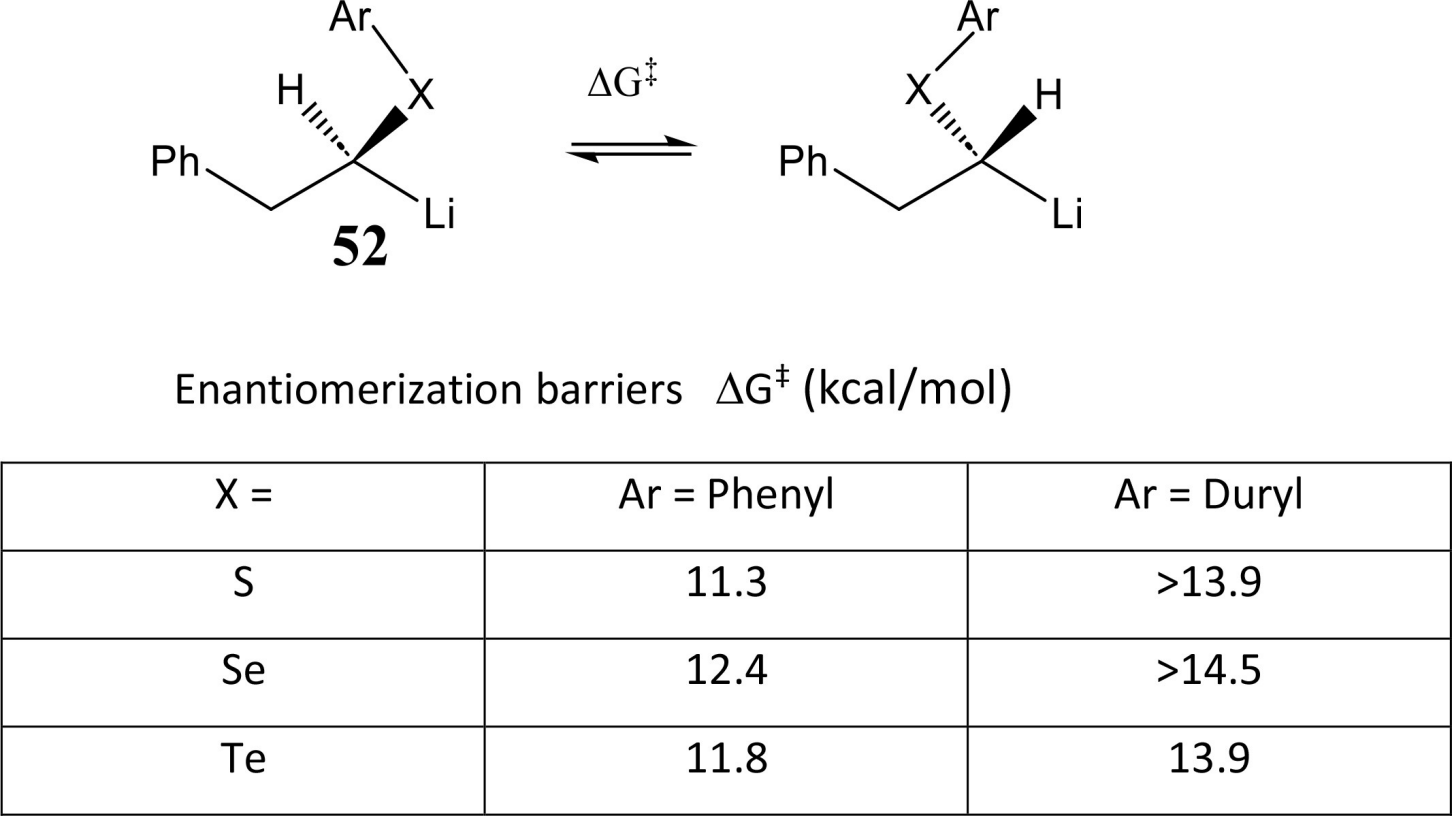
Enantiomerization barriers of α‐chalcogeno‐alkyl‐lithium compounds.

At this point, detailed information on the configurational stability of α‐hetero‐alkyllithium‐compounds became necessary. This led to a change of our research focus, away from the differentiation of diastereotopic groups (the means to generate α‐hetero‐alkyllithium‐compounds) to the α‐hetero‐alkyllithium‐compounds themselves. On studying the enantiomerization barriers in α‐hetero‐alkyllithium‐compounds **52** we found,^[67]^ that a duryl residue on the heteroatom, instead of a phenyl group, increased the enantiomerization barrier to the point that these α‐hetero‐alkyllithium‐compounds **52** can be considered as macroscopically configurationally stable at −100 °C: (Scheme 43)

**Scheme 44:**
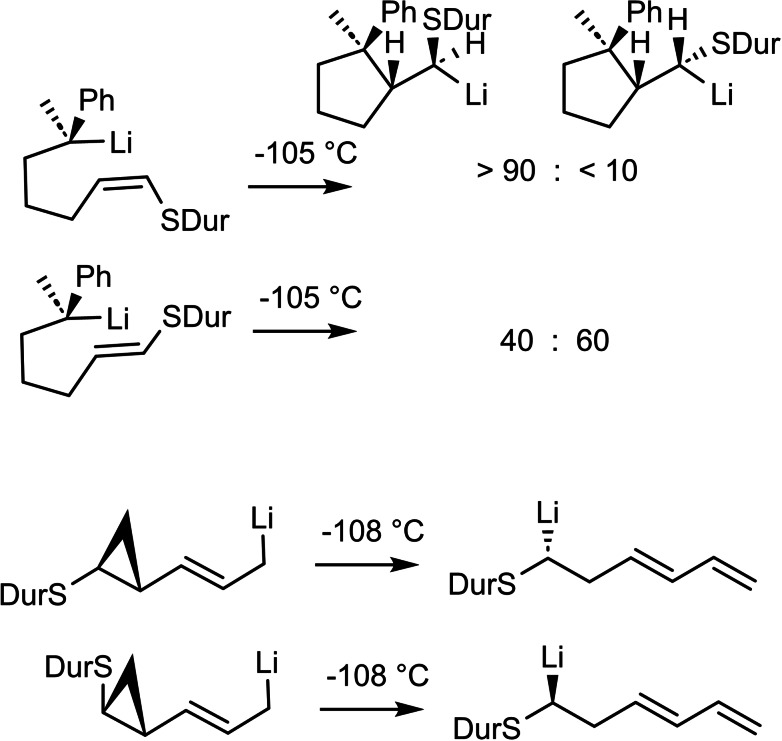
Stereochemistry of carbon‐lithium bond forming reactions.

These findings opened the opportunity to establish for the first time the stereochemistry of reactions, in which carbon‐lithium bonds are *being formed*, e. g. the carbolithiiation of vinylsulfides^[68]^ or the ring‐opening of cyclopropyl‐carbinyl‐lithium compounds.^[69]^ (Scheme 44) In the end this was a long journey from the initial efforts to find reactions that effectively differentiate diasterotopic groups.

## 7 Special Developments, The „Hoffmann“‐Test

**Scheme 45:**
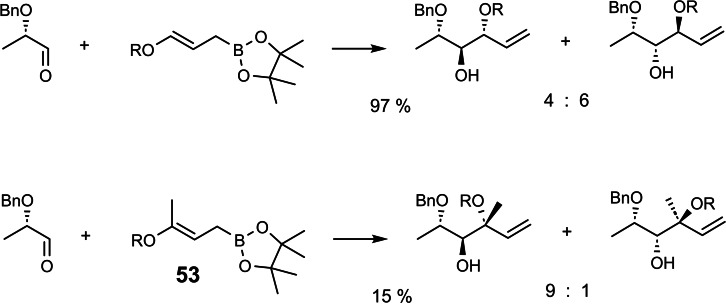
Allylboration with γ‐alkoxy‐allylboronates.

During the study of allylboronate additions, we investigated the addition of γ‐alkoxy‐allylboronates to protected lactaldehydes with the aim to realize a (C_3_ + C_3_)‐synthesis of 2,6‐dideoxy‐hexoses.^[70]^ (Scheme 45)

When extending the reaction to γ‐alkoxy‐crotylboronates **53** with the aim to generate 2,6‐dideoxy‐3‐methyl‐hexoses we realized good diastereoselectivity but met unacceptable low yields even when running the reactions under 8 kbar pressure. The dominance of side reactions signaled that the limits of this approach had been reached.

**Scheme 46:**
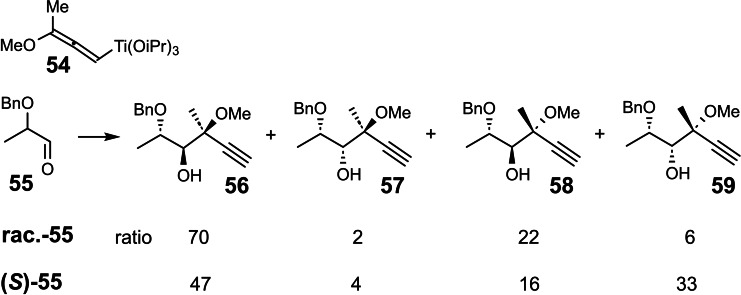
Extension of aldehydes with a γ‐alkoxy‐allenyl‐titanium reagent.

In search of alternatives we turned to the allenyl‐titanium compound **54**. Exploratory experiments with racemic lactaldehyde **55** resulted in 80 % of the diastereomeric adducts **56–59**, among which isomer **56** prevailed to 70 %. (Scheme 46)

This finding encouraged us to react the titanium compound **54** with the enantiomerically pure aldehyde **55**. While high yield in adduct formation was maintained, the diastereomer ratio of the adducts was markedly different.^[71]^ It took less than half an hour to see the solution to this puzzle: Not only the aldehyde **55**, but also the reagent **54** is chiral, yet as applied, a racemate! On reaction with the *enantiomerically pure* aldehyde **55**, one enantiomer of **54** leads to the products **56** and **57** (in together 51 %), whereas the other enantiomer of **54** leads to products **58** and **59** (combined 49 %). The 1 : 1 ratio of the product combinations corresponds to the 1 : 1 ratio of the enantiomers in the racemate of **54**. When however, the racemate of **54** is allowed to react with *racemic* aldehyde **55**, products **56+57** arise by kinetic resolution as the matched pair and faster reacting combination in a higher percentage than products **58** and **59** from the mismatched combination of **54** and **55**.

So much for the experimental results. A few weeks later, I was due to write a report for the German Science Foundation. I was concerned avoiding too many negative attributes to the above results. I realized that the above experiments demonstrated that the allenyl‐titanium compound **54** is configurationally stable on a microscopic time scale, defined by the rate of its addition to the aldehyde **55**. And, suddenly I was fully awake! The statement regarding configurational stability of **54** was derived using a *racemate*, and not an enantiomerically enriched or pure sample! Such a test could be applied to other chiral – but for convenience – *racemic* organometallic reagents. It all amounted to adding the racemate of X to a chiral aldehyde, once to the racemic aldehyde, once to the enantiomerically pure aldehyde, and simply to record the ratio of the diastereomeric products. Are the product distributions equal, the substrate X is found to be configurationally labile on the microscopic time scale, are they different, X would be found to be configurationally stable.

This conclusion, once thought, was irrevocably established, an infinitesimal progress of science. The underlying principle was so general, that I felt sure that someone must already have reported it with reference to some other system. I asked whatever colleagues I met at conferences, or visitors that came to my office. None of these was aware of any such precedent. Finally, after a year or so, I dared to publish these findings.^[72]^ I anticipated nasty letters of irate colleagues pointing out that I missed their fundamental contributions. Yet, as of today, no such letter came in.

**Scheme 47:**
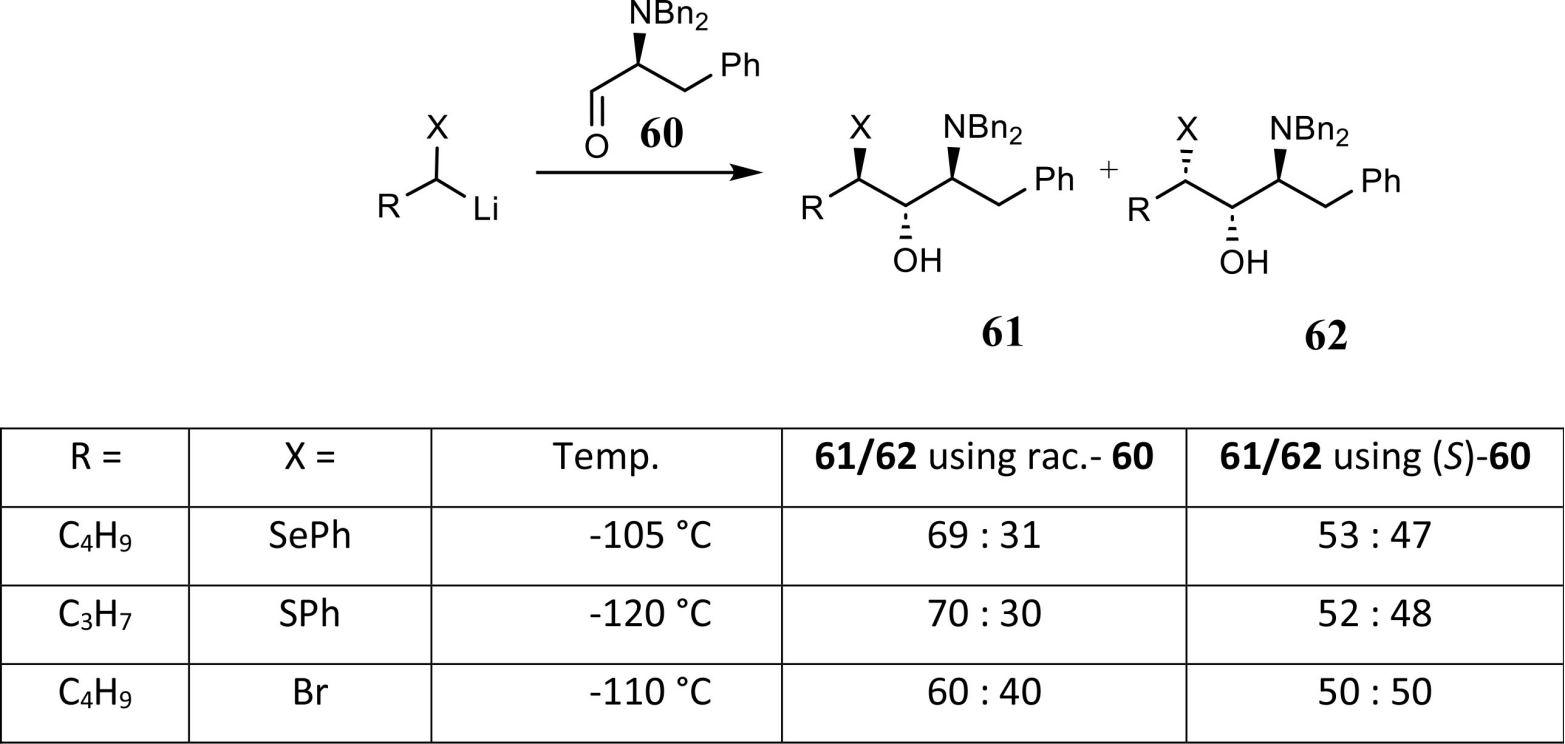
Test on the microscopic configurational stability of α‐heterosubstituted organolithium reagents.

We then defined the optimal conditions for carrying out such tests^[73]^ and tested the α‐heterosubstituted alkyllithium compounds of interest to us.^[74]^ (Scheme 47) We found that for X=SePh, SPh, Br all compounds racemized more slowly than they added to the chiral aldehyde **60**. The test offers only a qualitative statement, though, which is all right to judge conditions for preparative work. More quantitative statements are not simply available, since they are based on a comparison of a monomolecular rate of racemization with a bimolecular, and hence, concentration dependent rate of addition to a chiral trapping agent.

**Scheme 48:**
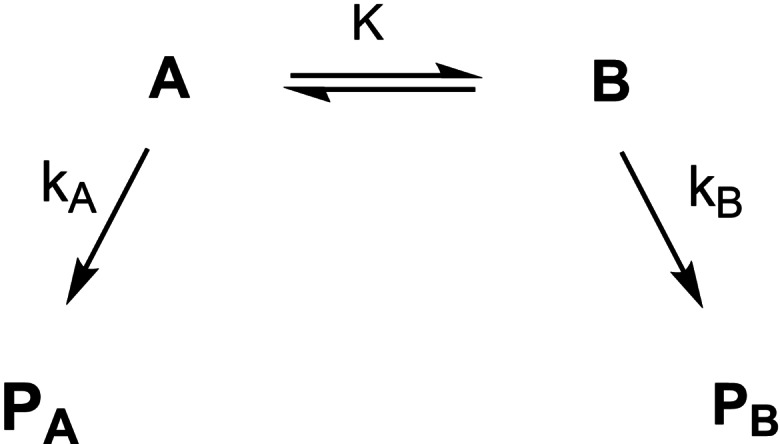
Product formation in a Curtin/Hammett situation.

The test has been readily adapted by the chemical community. A compilation of results and variants of the test can be found in ref. ^[75]^. The test can be applied to analyze systems of the following kind related to the Curtin/Hammett schemes:^[76]^ (Scheme 48)

The compounds **A** and **B** do not have to be symmetry‐related (enantiomers). They could be diastereomers or different compounds. The equilibrium constant should be 0.2<K<5. The products **P_A_** and **P_B_** should be constitutionally and configurationally stable and should carry a signature that reveals their origin from either **A** or **B**. The conversion of **A** and **B** into **P** should be irreversible and the rates k_A_ and k_B_ should differ in the range k_A_/k_B_≈1.5 to 3.

**Scheme 49:**
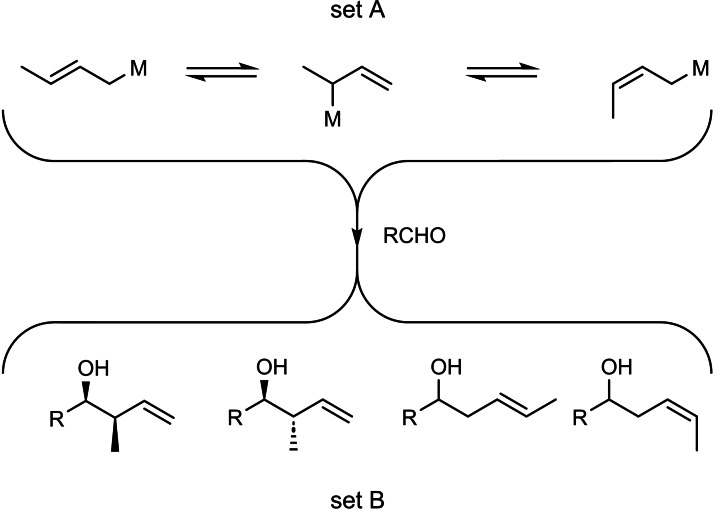
Test on the configurational stability of η^1^‐allyl‐metal compounds.

We realized that this scenario would allow gathering information on the metallotropic rearrangement of η^1^‐crotyl‐metal‐compounds:

Such metallotropic rearrangements screw up the application of η^1^‐crotyl‐metal‐compounds in synthesis, when one intends to convert a distinct member of set A into a distinct member of set B. The critical question is, whether the mutual interconversion of the members of set A is faster or slower than their conversion to individual members of set B by addition to an aldehyde. (Scheme 49)

**Scheme 50:**
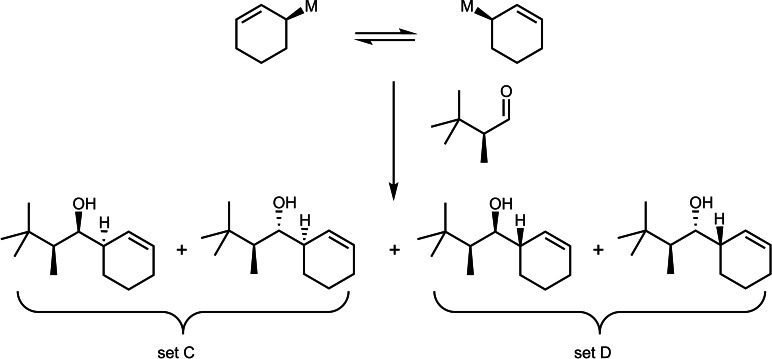
Test on the configurational stability of η^1^‐allyl‐metal compounds.

This situation could be illuminated by the following test system for M=Li, MgCl, Ti(O^i^Pr)_3_, B(OR)_2_, BEt_2_ by determining the product set C/set D ratios. It was found that metallotropic shifts were only with the boron‐derivatives slower than the addition to the applied aldehyde.^[77]^ (Scheme 50)

In hindsight, we were lucky to recognize the opportunities opened by the unexpected outcome of the addition of the allenyl‐titanium compound **54** to the enantiomerically pure aldehyde **55**. This reminded me of a sentence by Walter Hückel in the obituary for H. Kiliani:^[78]^

*Hier ging Kiliani an dem Tor vorbei*, *durch das wenig später Emil Fischer zur Stereochemie der Zucker gelangte. Kiliani drehte an ihm mit seinen Experimenten den Schlüssel zweimal herum und schloss es damit wieder zu*, *während er nur einmal hätte zu drehen brauchen*, *um es zu öffnen*.

In the above case, I had recognized that there was a door to be opened. However, in how many other instances did I fail to recognize that I held a door‐handle in my hand?

## 8 α‐Heterosubstituted Alkylmagnesium‐Compounds

**Scheme 51:**
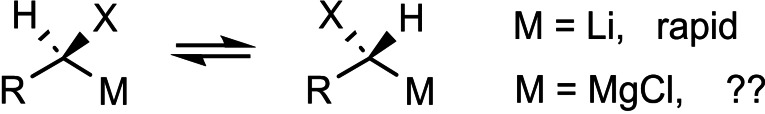
Racemisation of α‐heterosubstituted organometal compounds.

As shown before, α‐heterosubstituted alkyllithium compounds possess a high tendency for enantiomer‐equilibration.^[79]^ We hoped that the corresponding magnesium compounds would display considerable higher configurational stability. (Scheme 51)

**Scheme 52:**
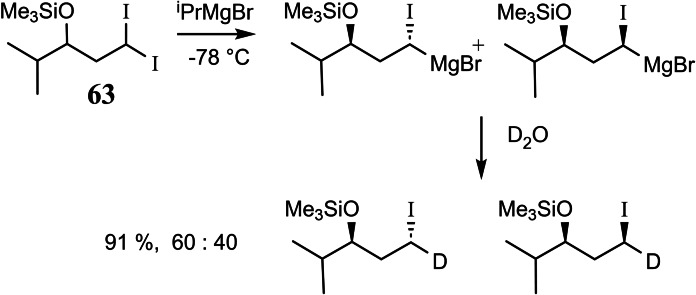
Iodine/magnesium exchange on diastereotopic iodine atoms.

To clarify this point, we turned – in continuation of earlier studies^[80]^ – to an investigation of a diastereoselective iodine/magnesium‐exchange at compound **63**. (Scheme 52)

**Scheme 53:**
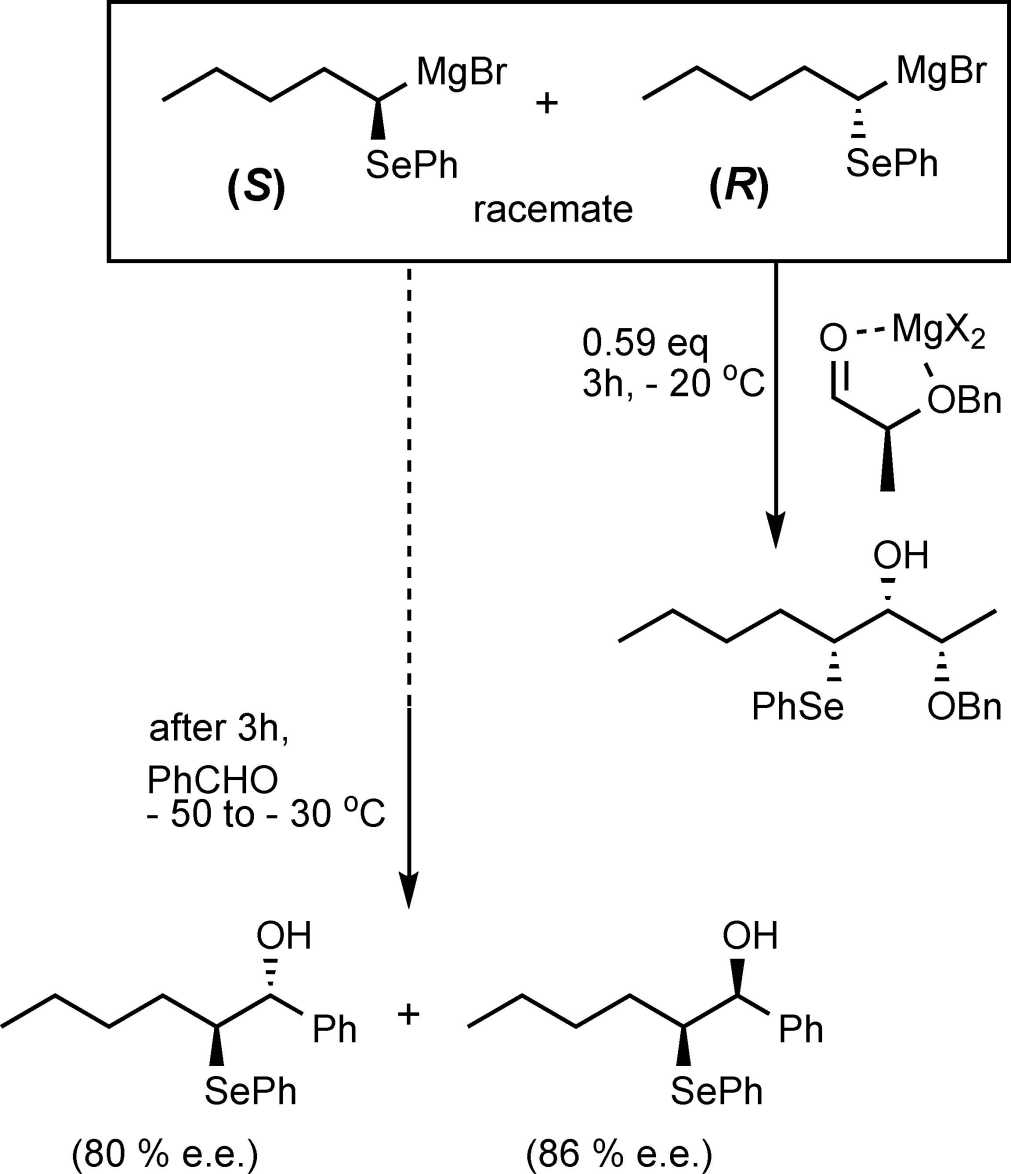
Kinetic resolution of an α‐phenylseleno‐alkyl‐magnesium compound (Adapted from Chem. Ber. **1996**, 129, 633 with permission from John Wiley and Sons).

The resulting magnesium‐carbenoids could be generated in good yield and turned out to be configurationally stable up to ca. −20 °C.^[81]^ The low diastereoselectivity in this reaction though precluded further application in stereoselective synthesis. Nevertheless we succeeded subsequently in a kinetic resolution of an α‐phenylseleno‐alkyl‐magnesium compound,^[82]^ which could be manipulated at temperatures <−20 °C without racemization. (Scheme 53)

**Scheme 54:**
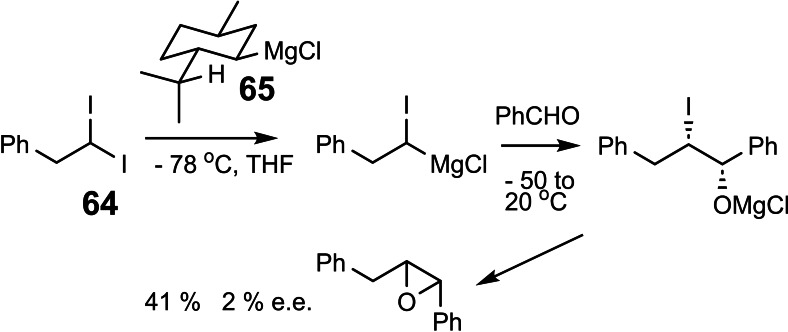
Attempted kinetic resolution of an 1,1‐diiodoalkane.

Both these findings led us to search with high priority for a simple way to generate enantiomerically pure α‐halogeno‐alkyl Grignard reagents. Initially we hoped to attain this goal by a differentiation of the enantiotopic iodine atoms in compound **64**, a differentiation to be realized by a chiral Grignard reagent. However, using menthyl Grignard **65**, the level of asymmetric induction remained vanishing small.^[83]^ (Scheme 54)

**Scheme 55:**
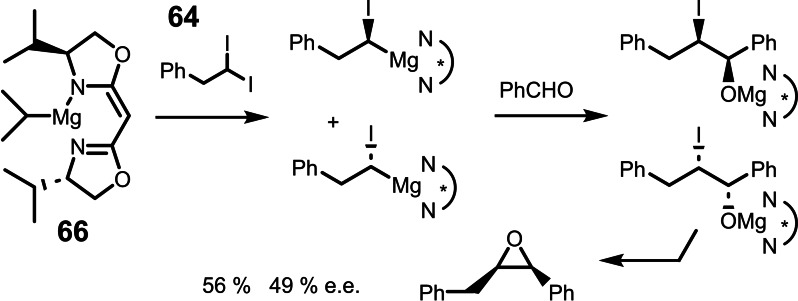
Attempted kinetic resolution of an 1,1‐diiodoalkane.

In reagent **65**, the chiral information is part of the alkyl part of the Grignard reagent (the nucleophilic part). A different situation holds for the reagent **66**, in which the electrophilic part (the magnesium cation) carries the chiral modification. Using reagent **66** alas, a substantial asymmetric induction could be realized: (Scheme 55)

**Scheme 56:**
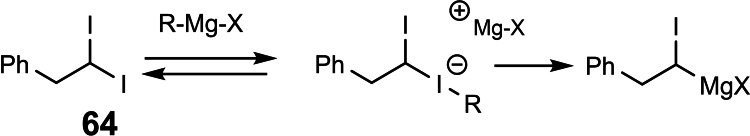
Proposed mechanism of the iodine/magnesium exchange reaction.

The different outcome of these two reactions gave unsolicited hints as to the mechanism of the iodine/magnesium exchange on 1,1‐diiodoalkanes, to the point that the first step – in which the nucleophilic property of the Grignard reagent counts – appears to be fast and reversible, whereas the second step – in which the electrophilic properties of the Grignard reagent matter – would be rate‐ and product‐determining. (Scheme 56)

Yet, these remarks are nothing but whitewashing of the situation that 49 % e.e. is an achievement but does not provide a solution of the task.

**Scheme 57:**
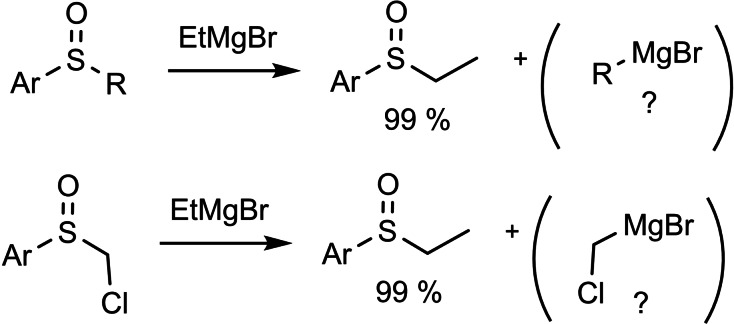
Sulfoxide/magnesium exchange reaction.

We knew from our previous engagement with sulfoxide chemistry, that the alkyl residue in aryl‐alkyl sulfoxides can be exchanged by alkyl‐Grignard reagents, a reaction commonly used to synthesize distinct aryl‐alkyl‐sulfoxides. The fate of the formal co‐product, a new Grignard reagent, had never been examined. (Scheme 57)

Even, the substitution of a chloromethyl residue had been reported^[84]^ yet no information on the subsequent fate of this residue was given. We reasoned, that α‐chloro‐sulfoxides, easily accessible diastereomerically and enantiomerically pure, could provide access to the desired α‐chloroalkyl‐magnesium compounds. The stereochemistry of this transformation was known for the sulfur atom, but not for the replaced carbon atom.

**Scheme 58:**
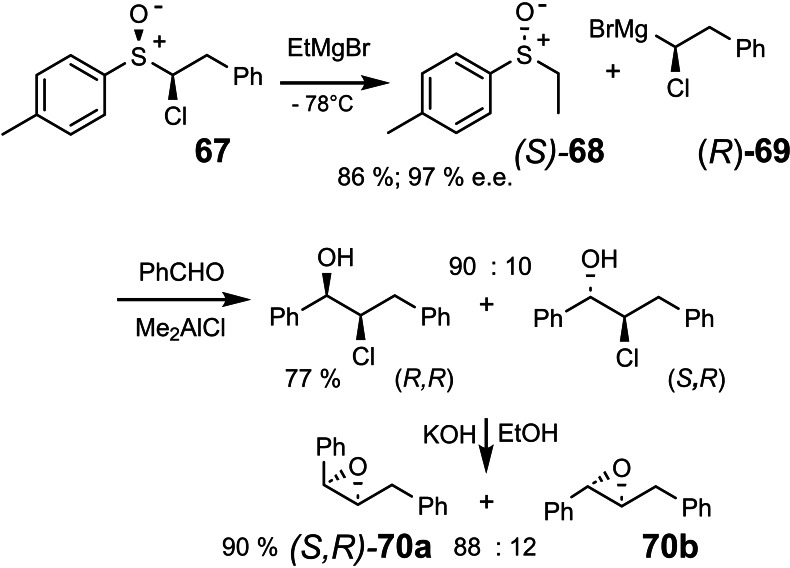
Sulfoxide/magnesium exchange to generate enantiomerically enriched α‐chloroalkyl‐magnesium bromide.

Peter Nell generated in few steps enantiomerically and diastereomerically pure sulfoxide **67**. Its reaction with ethyl‐Grignard provided as expected the sulfoxide **68** in high enantiomeric purity. (Scheme 58)

The sought after carbenoid **69** could be trapped in the reaction medium by benzaldehyde to furnish the chlorohydrins, which were characterized as the epoxides **70**. Epoxide **70 a** was obtained in an enantiomeric purity of 93 % e.e. proving that we established a route to practically enantiomerically pure Grignard reagent **69**!

**Scheme 59:**
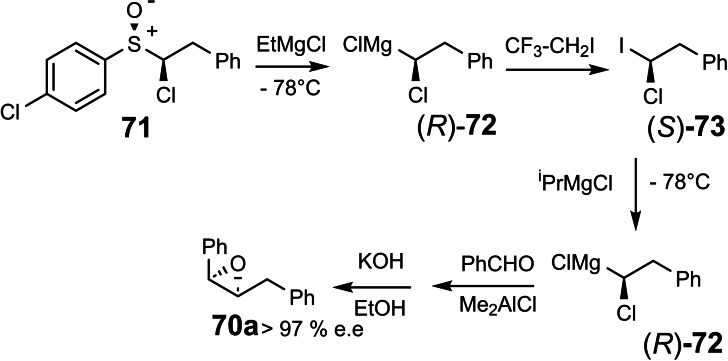
Iodine/magnesium exchange to generate enantiomerically enriched α‐chloroalkyl‐magnesium chloride.

With the absolute configuration of **67** and **70 a** known, it followed that the sulfoxide/magnesium exchange had occurred with retention of configuration at the replaced carbon atom.^[85]^ In due course, further stereochemical relationships could be established: Enantiomerically pure sulfoxide **71** led via (R)‐**72** to the chloro‐iodo‐alkane **73**, on which the iodine/magnesium‐exchange could be effected with complete retention of configuration at the metal‐bearing carbon atom.^[85]^ (Scheme 59)

Before studying the stereochemistry of reactions of the chiral carbenoids **69**, respectively **72**, it was mandatory to establish the limits of their configurational stability. Carbenoid **72** was found to be configurationally stable up to ≤−20 °C. Halide anions, especially bromide ions, however lead already at −50 °C to racemization, probably by a S_N_2‐type nucleophilic halide exchange.

**Scheme 60:**
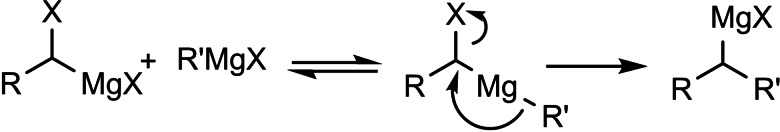
Homologation of α‐haloalkyl‐magnesium compounds.

The interest in chiral magnesium‐carbenoids relates to the homologation reaction developed by Villieras:^[88]^ (Scheme 60)

**Scheme 61:**
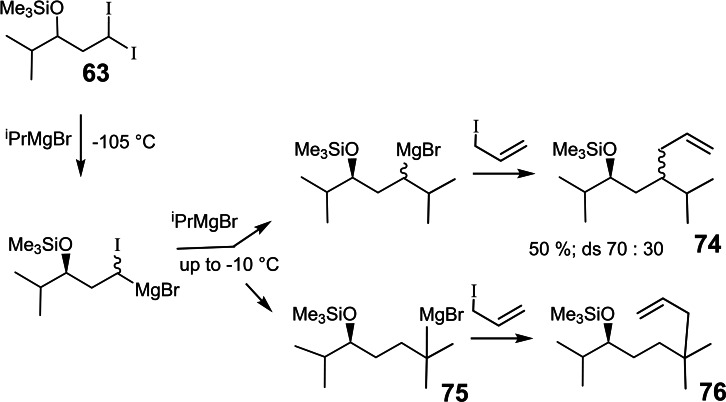
Homologation of α‐haloalkyl‐magnesium compounds to give secondary alkyl‐Grignard reagents.

Could chiral magnesium carbenoids be used to generate chiral (secondary) Grignard reagents, hitherto inaccessible? For this reason, we checked the homologation reaction already during the first experiments with the di‐iodo compound **63**. (Scheme 61)

The homologation reaction could be realized as expected. The diastereomer ratio of the products **74** conformed to expectations. Andreas Kusche though, noted that another product **76**, isomeric with **74** had been formed in 4 % yield. When the solvent was changed from THF to ^t^BuOMe, compound **76** turned out to be the main product in 58 % yield.^[87]^ The unexpected generation of a tertiary Grignard reagent **75** has since been substantiated using other substrates than compound **63**, and opened a skeleton‐expanding route to tertiary Grignard reagents.^[88]^

**Scheme 62:**
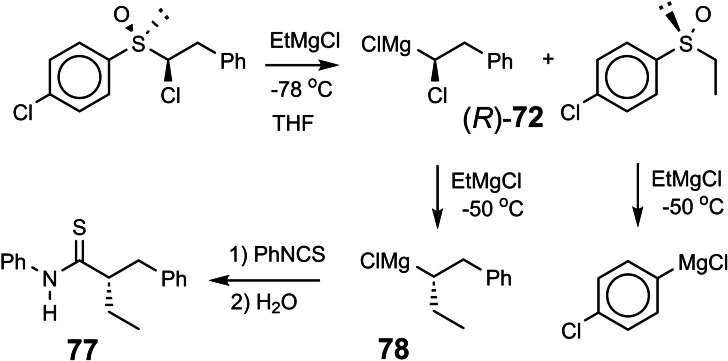
Homologation of α‐haloalkyl‐magnesium compounds to give enantiomerically enriched secondary alkyl‐Grignard reagents.

These studies, importantly, defined conditions for a clean homologation reaction of magnesium carbenoids. This allowed then to access by „synthesis“ for the first time a chiral secondary Grignard reagent **78**, in which the metal‐bearing carbon is the only element of chirality:^[89]^ (Scheme 62)

The thioamide **77**, obtained at the end of the reactions sequence, had an enantiomeric purity of 93 %. Knowledge of the absolute configuration of **72** and of **77** allowed the conclusion that the homologation of **72** to **78** had occurred with inversion of configuration at the magnesium‐bearing carbon atom.^[89]^ Grignard reagent **78** of 97 % e.e. racemized at −10 °C under the reaction conditions with a half‐life of 5 h.

Even when **78** could not be generated in a pure state, but only in a cocktail containing excess EtMgCl and coproduct ClC_6_H_4_MgCl, it allowed to gain fundamental informations on the reaction of Grignard reagents with electrophiles.^[90]^ When an electrophile attacks a Grignard reagent at the metal‐bearing carbon atom according to a S_E_2‐mechanism, the product should be enantiomerically pure (or enriched). When however, the reaction is triggered by an one‐electron transfer from the Grignard reagent to the electrophile, the latter would generate a sec.‐alkyl radical with an enantiomerization barrier of <1,5 kcal. The products obtained would hence be generated essentially as racemates.

With the solutions of **78** we could test a wealth of standard Grignard reactions in this regard.^[90]^

**Scheme 63:**
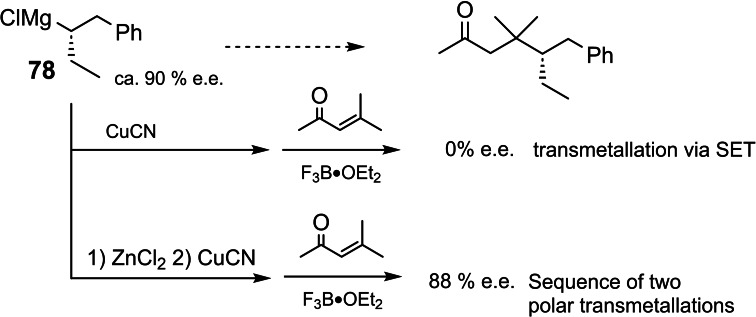
Transmetallation from magnesium to copper(I).

We present here only a surprising observation on the transmetallation of Grignard reagent **78** to Zn^++^, resp. Cu^+^.^[91]^ (Scheme 63)

It transpires that on transmetallation of **78** to Cu^+^, the oxidation potential of Cu^+^ suffices to oxidize **78** to the corresponding radical. On transmetallation of **78** to Zn^++^, the latter is so weak an oxidant that a direct transmetallation to the organozinc reagent is effected. The latter, though is not as easy to oxidize as the precursor Grignard reagent **78**. A subsequent transmetallation to Cu^+^ could accordingly be realized without a competing one‐electron transfer.

At the outset, we had the hope that α‐haloalkyl‐magnesium reagents (magnesium carbenoids) might have a configurational stability sufficient to allow their application in stereoselective synthesis. This tenet has been fully substantiated and led to numerous further insights into the stereochemistry of organometallic reactions.

## 9 Special Developments, Iodine‐Ate‐Complexes

**Scheme 64:**
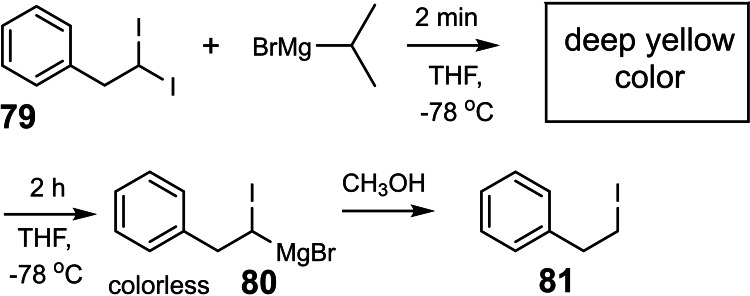
Color phenomenon on iodine/magnesium exchange.

During our initial studies on magnesium carbenoids (Section 7) Phil Meister added isopropyl‐magnesium bromide to a solution of the diiodo‐alkane **79** at −78 °C. He noted an immediate pumpkin‐yellow coloration of the solution that faded away over half an hour. Thereafter the carbenoid **80** could be trapped in diverse reactions. The spectacular color phenomenon could be consistently observed in all subsequent experiments. (Scheme 64)

**Figure 3:**
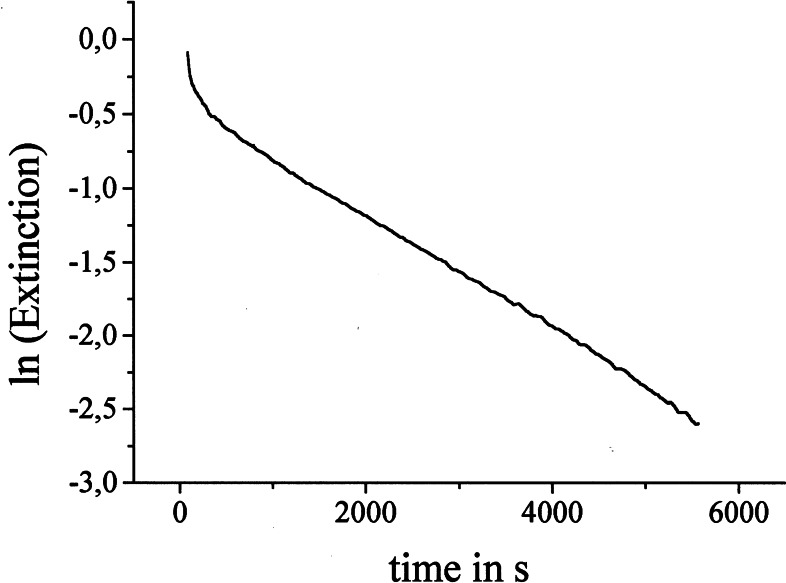
Rate of decolorization on iodine/magnesium exchange, Reprinted with permission from Organometallics **22**, 2925. Copyright 2003 American Chemical Society.

We were puzzled by this color phenomenon, but I hesitated for long to assign the clarification of it as a project to a graduate student, as I knew the risk of a complete failure. Eventually in 1996, Mark Brönstrup asked me for a real challenge and he did not hesitate to tackle this problem. He recorded, that the color faded with a first order reaction rate, and that the pseudo‐first order rate constants were proportional to the [Mg^++^]‐concentration. These rate constants were the sole characteristic number for the colored species. (Figure 3)

**Scheme 65:**
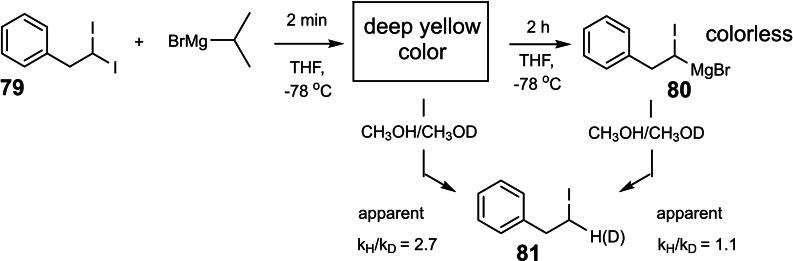
Isotope effects to distinguish intermediates in iodine/magnesium exchange.

**Figure 4:**
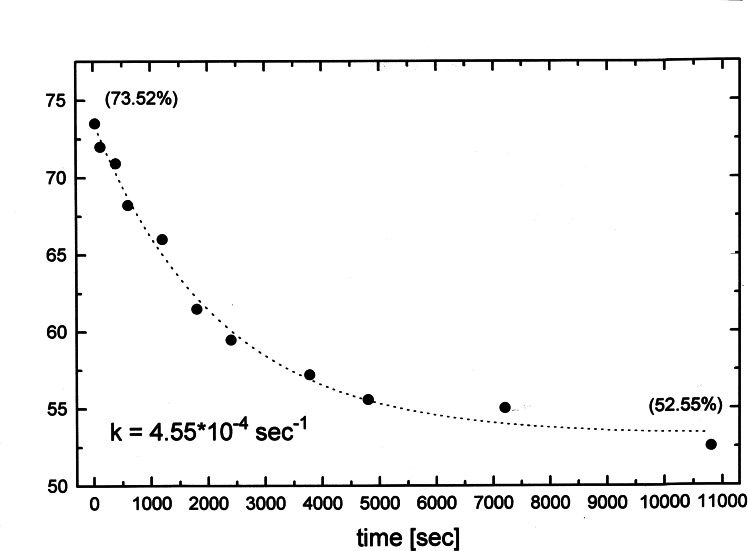
Change of the apparent isotope effect over time on iodine/magnesium exchange. Reprinted with permission from Organometallics **22**, 2925. Copyright 2003 American Chemical Society.

Volker P.W. Böhm continued the investigation and found that on quenching of the deep yellow solution with methanol the same iodo‐alkane **81** was obtained as on quenching the decolorized solution. Provided it is present in appreciable concentration, the colored species will thus give the same protonation product as the carbenoid **80**. A distinction appeared possible with reference to a competition constant, e. g. between protonation and deuteration. (Scheme 65) And that was the case:

The clou was found, when Volker Böhm determined the time dependence of the apparent isotope effect. It changed with the same rate constant, as the disappearance of the yellow color: (Figure 4)

**Figure 5:**
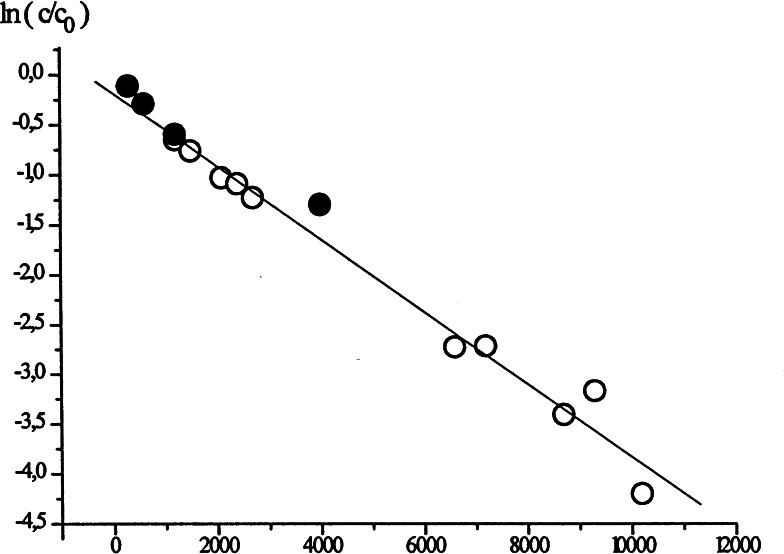
Rate of changes in nmr signal intensities on iodine/magnesium exchange. Solid and open circles refer to the time‐course of different signals of the intermediate Reprinted with permission from Organometallics **22**, 2925. Copyright 2003 American Chemical Society.

This established a connection between the colored species and the one that gave rise to the higher selectivity on protonation/deuteration. Finally, Michael Müller recorded under rapid injection conditions low temperature ^13^C‐ and ^1^H‐nmr‐spectra of the yellow solution and found signals that disappeared with time. (Figure 5)

Again, the signal intensities decreased with the same rate constant as that of the disappearance of the yellow color. These signals could therefore be attributed to the yellow species, which must accordingly be present in stoichiometric amounts in solution.

**Scheme 66:**
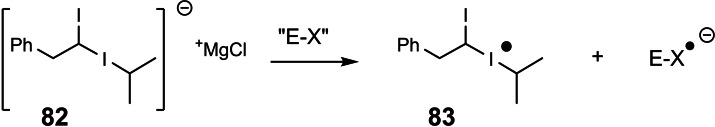
Induced decolorization of the „iodine ate.complex”.

Interpretation of these and further results suggested that the colored moiety is the iodine‐ate‐complex **82**. Iodine ate‐complexes have repeatedly been discussed as short‐lived intermediates in iodine‐metal exchange reactions. In the case of **82**, the second iodine atom might exert sufficient stabilization to render the particular ate‐complex long‐lived and observable.^[92]^ (Scheme 66)

This lucky situation opened the possibility to study the chemistry germane to iodine‐ate‐complexes with **82** as prototype. In doing so, we noted that surprisingly the conversion of **82** to the carbenoid **80** was triggered by a variety of widely differing substances (benzaldehyde, carbon tetrachloride, tosyl chloride, nitromethane). The common feature of these „catalysts“ is, that they are amenable to facile one‐electron reduction. The ate‐complex **82** in turn, having electrons in a non‐bonding or anti‐bonding molecular orbital, should be readily oxidized to a dialkyl‐iodinane‐radical **83**. The latter should immediately loose an alkyl radical, which could initiate a radical chain to convert the ate complex **82** into the carbenoid **80**.^[93]^ The depicted scenario remains at present mere speculation. Nevertheless, it provides a reasonable explanation for the repeated observation of products derived from free radicals in halogen/metal exchange reactions.

This study was initiated by an unexpected observation of a color phenomenon. It led in the end to novel insights into the stability and chemical behavior of iodine‐ate‐complexes. According to the Science Citation Index this study may be rated as rather insignificant for the chemical community. On the other side, I deem this study the probably intellectually best scientific piece that originated from our research group.

## 10 Transition State Conformations

Initially stereoselective synthesis comprised hardly anything more than a part‐discipline of steroid chemistry. During the 1970s and 1980s of the past century stereoselective synthesis mutated into a main topic in organic chemistry. The pertinent knowledge had been compiled in 1971 into a text of 450 pages.^[94]^ Ten years later, a compilation filled two volumes with together 850 pages,^[95]^ eventually by the mid of the nineties it filled six volumes with close to 7000 pages.^[96]^ To keep up with such a dramatic development was difficult for outsiders and definitely a challenge for academic staff.

Accordingly, the pharma research department of Bayer AG asked me in 1987 for a two‐day seminar, in which I covered simple diastereoselectivity. Subsequently, Bayer asked me for a succession seminar on asymmetric induction. That put me into a dilemma. The hundreds of publications addressing asymmetric induction corresponded to the situation that outsiders maintain of organic chemistry: A big pile of unrelated facts without any helpful connections. On reading these papers, I had, in order to maintain an unbiased approach, intentionally ignored the tentative interpretations of the causes for the reported asymmetric inductions.

**Scheme 67:**
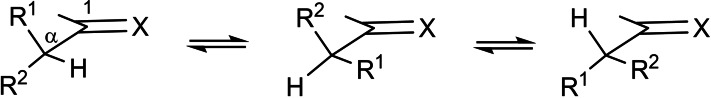
Rotation around the bond separating the present stereocenter and the prochiral reaction center.

Now, I could no longer avoid a discussion of why certain reactions led to high asymmetric induction. Most of the stereogenic reactions were additions to planar prochiral groups (C=X; X=CR_2_, NR, O). When the substrate contained already a stereogenic center, frequently in α‐position, upper and lower face of the planar reaction center are diastereotopic. The extent of asymmetric induction exerted by the stereocenter on the addition in the two diastereotopic half‐spaces, depends on the nature of the residues R^1^, R^2^, and H, and prominently, on the conformation at the C‐1−C‐α‐bond in the transition state of the addition reaction. (Scheme 67)

The role played by the residues R^1^, R^2^ has been characterized by Winterfeldt uniquely as *active* or *passive volume*,^[97]^ – a misnomer, one couldn't do without though. The role of the conformation at the C‐1−C‐α‐bond is to bring the active or passive volumina of R^1^ and R^2^ into action. It remained, however, speculation, which transition state conformation were dominant in which reactions. There existed frequently different models for a given reaction, which assigned different roles to the residues R^1^ and R^2^, and hence postulated different transition state conformations, which happened to be mutually exclusive.

**Scheme 68:**
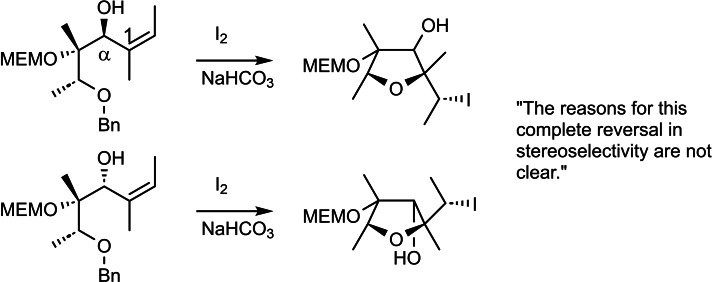
Asymmetric induction in intramolecular iodo‐etherification.

Such was the situation when I started to fit individual observations into an overarching scheme on preparation for the lecture course. In doing so, I came across the following report regarding an iodo‐etherification,^[98]^ (Scheme 68) in which I felt challenged by the comment of the authors.

**Scheme 69:**
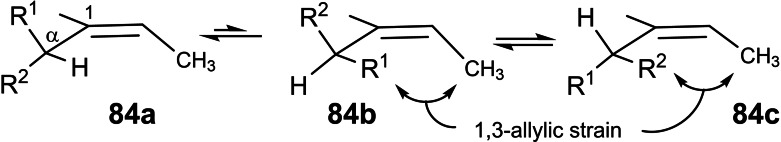
Rotation around the bond separating the present stereocenter and the prochiral reaction center.

In the present case, the asymmetric induction appears to originate solely from the allylic stereogenic center. It carries an OH‐group, seemingly a passive volume, and the benzyloxy‐ethyl residue as active volume. Swapping of the two passive volumes OH and H will alter the sense of asymmetric induction only when this changes the conformation at the C‐1−C‐α‐bond. I fortunately recognized, that the presence of the Z‐positioned methyl group at the double bond allows in each case only for a single conformation that is free of 1,3‐allylic strain.^[99]^ It is this very conformation that accounts for the formation of the product in each instance. (Scheme 69)

**Figure 6:**
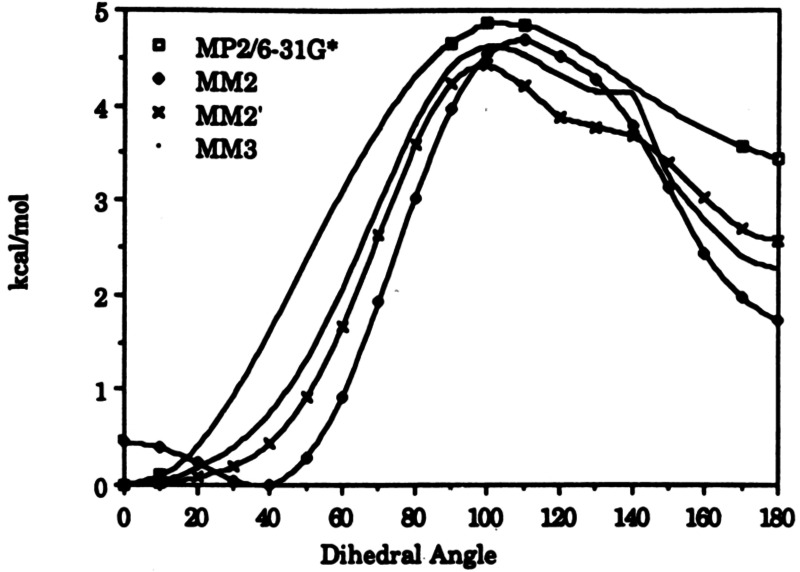
Rotational potential for the C‐3/C‐4 bond rotation in cis‐4.methyl‐2‐pentene, corresponding to C‐1/C‐α in **84**. Reprinted with permission from J. Amer. Chem. Soc. **113**, 5006 Copyright 1991 American Chemical Society.

That solved the problem at hand. As Ken Houk was at that time a visitor in Marburg, I asked him, whether he could calculate the rotation potential at the C‐1−C‐α‐bond in compound **84**. The resulting diagram (Figure 6) disclosed, that conformation **84 a** is by ca. 3.4 kcal/mole more stable than the conformations **84 b** and **84 c**, which are destabilized by 1,3‐allylic strain.[^100^, ^101^]

During the collection of the examples for the course, I hit several more cases, where the avoidance of conformations with 1,3‐allylic strain appeared as key for high asymmetric induction in addition reactions. In the end, they added up to a respectable list of references.

The year 1987 had come to an end. In the next spring I spent two months with a JSPS‐fellowship in Japan. I spent Easter time at the RIKEN institute in Wako in the Tokyo metropolitan area. My hosts apologized for being absent, attending a conference. Yet they left me with an access to the outstanding library of the RIKEN institute, which harbored everything of interest to an organic chemist and beyond. I started early in the morning drafting the manuscript on 1,3‐allylic strain as a control element in stereoselective synthesis. I continued till late evening, interrupted only by a short stroll to pick up some food. After four paradisiac days, the draft of the manuscript was completed and was submitted to Chemical Reviews after my return to Marburg.^[102]^

This review turned into a citation classic, which up to today has been quoted more than 1300 times. The review arose by a rather fortunate combination: I was aware of the concept of 1,3‐allylic strain^[6]^ when pondering about the problematic iodo‐etherification reaction. The explanation based on the concept of 1,3‐allylic strain was present in my mind, when scanning diverse reactions in preparation of the notes for the course on asymmetric induction. Only in this constellation did the general significance of this phenomenon become obvious to me.

## 11 Pederin and the Mycalamides

**Scheme 70:**
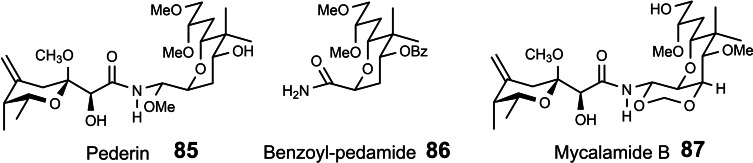
Pederin and mycalamide natural products.

**Scheme 71:**
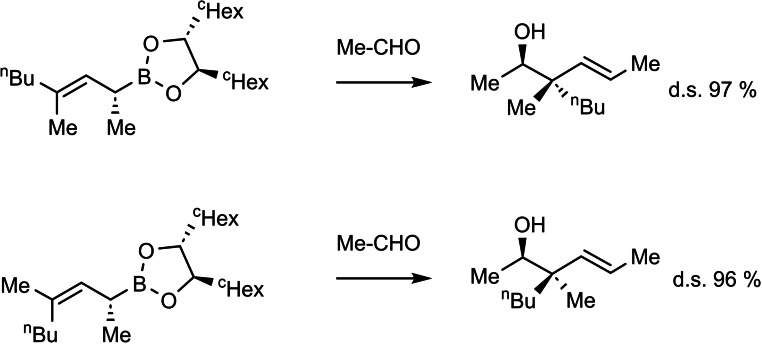
Allylboration to forge quaternary stereocenters.

Our interest in pederin **85** (Scheme 70) arose during our investigations on allylboration reactions, cf. section 4. when we studied α,γ,γ‐trisubstuted allylboronates. On addition to aldehydes they allowed the generation of homoallylalcohols with chiral quaternary carbon centers in a highly enantioselective manner.^[103]^ (Scheme 71)

**Scheme 72:**
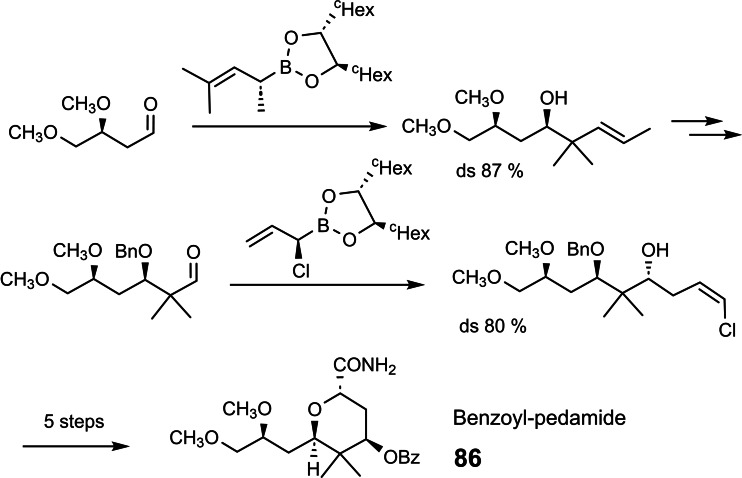
Synthesis of benzoylpedamide.

To demonstrate the value of this reaction and of allylboration in general, we looked for a target compound of current interest to the chemical community. We turned to benzoylpedamide **86**, a key intermediate in the synthesis of pederin **85**.^[104]^ In our ensuing synthesis of benzoylpedamide we enlisted two different allylboration reactions to set two of the four stereocenters with good reagent‐controlled diastereoselectivity:^[105]^ (Scheme 72)

As we were newcomers to the field of pederin/mycalamides, our contribution was meaningful only, when it was more efficient than previously recorded syntheses. Our synthesis happened to be just two steps shorter, essentially a runner‐up.

**Scheme 73:**
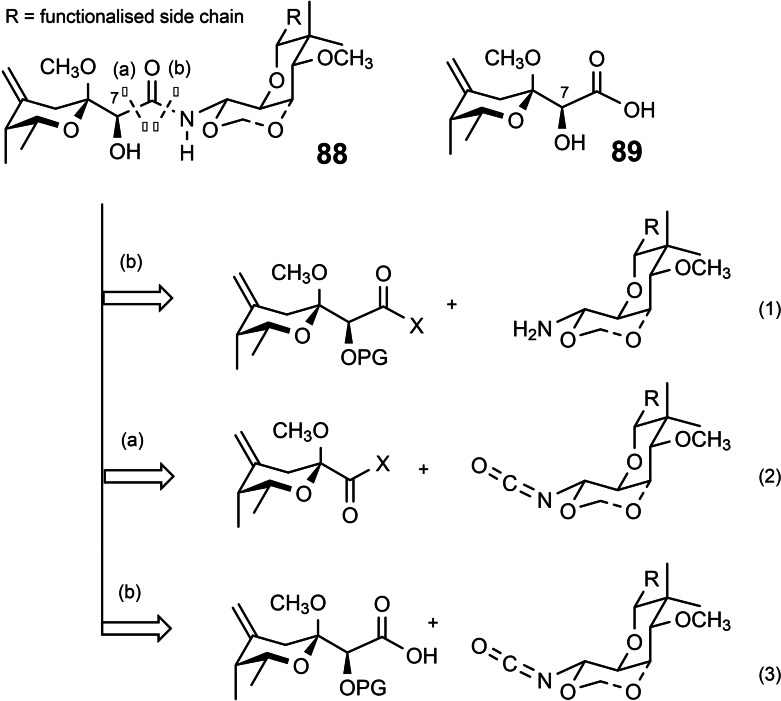
Retrosynthesis considerations for pederin/mycalamides. Reprinted with permission from Helv.Chim.Acta 2004, **87**, 1202, Copyright John Wiley and Sons.

By evaluating the then existing syntheses of pederin, as well as those of the related mycalamides **87**, we recognized the genuine synthetic challenges associated with these target structures. They relate to the combination of two obvious components – the right and left halves ‐, and to set the relative configuration of the half‐aminal stereocenter simultaneously.^[106]^ (Scheme 73)

Unfortunately, the simple half‐aminal moiety R‐CH(OR’)NH_2_ is stereochemically labile, such that routes (1) via such an entity invariably led to diastereomeric product mixtures. Enticed by such challenges we turned from “applications of allylboration reactions” to the specific project “total synthesis of pederin and mycalamide”, a broader project that does not necessarily involve allylboration.

**Scheme 74:**
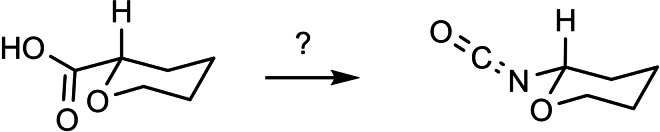
α‐Alkoxy‐alkylisocyanates by Curtius degradation.

We looked for a stereochemically stable compound as surrogate for the half‐aminal, R‐CH(OR’)NH_2,_ an intermediate that may be generated in a stereocontrolled fashion. We thus focused on the Curtius degradation of α‐alkoxy‐carboxylic acids to an α‐alkoxy‐alkyl isocyanate. (Scheme 74)

**Scheme 75:**
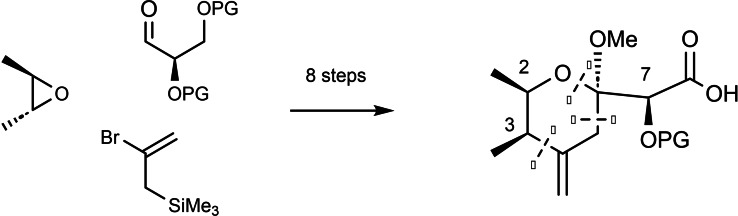
Building blocks for the synthesis of a protected pederic acid.

The isocyanate group should then directly serve in the combination of the two subunits. Regarding the joining of the two substructures, this opened further retrosynthetic disconnections (2) and (3) beyond the standard one (1) (Scheme 73). Pursuing this goal, required access to both constituting building blocks, a protected pederic acid **89** (left part) and an isocyanate modified right part. Concerning pederic acid, we utililized an approach (3) delineated in Scheme 73,^[106]^ (Scheme 75)

**Scheme 76:**
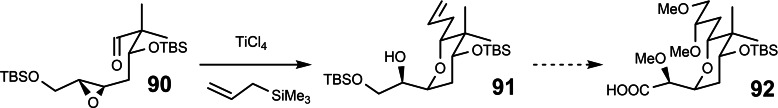
En route to the right part of pederin.

To obtain an α‐alkoxyacid **92** related to the right part of pederin, the epoxyaldehyde **90** was advanced to the pyran derivative **91**, having all stereocenters in correct configuration.^[107]^ (Scheme 76). The final four steps to **92**, remaining postponed as standard transformations.

**Scheme 77:**
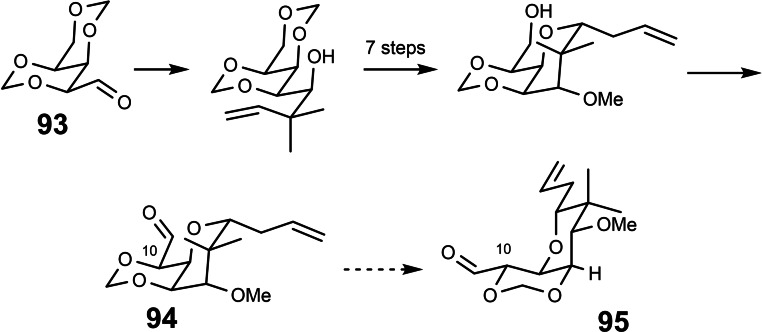
En route to the right part of mycalamide.

Regarding the right half of the mycalamides **87**, our initial approach was based on the readily available D‐sorbitol‐derived building block **93**. This choice, however, necessitated a later epimerization at C‐10, viz. **94** to **95**. (Scheme 77), which comprises a ring‐inversion of the trioxa‐cis‐decalin backbone.^[108]^

This epimerization failed at both the aldehyde and ester level, because – as we established later – the equilibrium lies, counter to our plans, on the side of **94**.

**Scheme 78:**
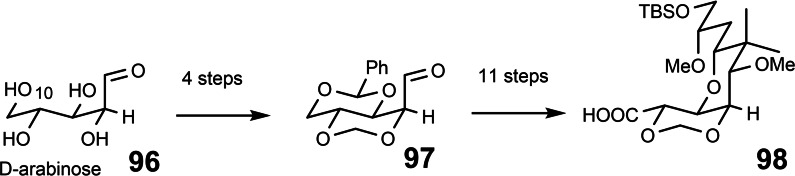
En route to the right part of mycalamide.

This situation necessitated a restart from another sugar precursor **96**, which would provide C‐10 with the correct absolute configuration. This condition was met by D‐arabinose. It readily yielded the key aldehyde **97**, which allowed the subsequent elaboration to the right‐hand building block **98** of mycalamide B.^[108]^ (Scheme 78) Thus, in retrospect, our enthusiastic choice of starting point **93** for the initial effort proved as overhasty.

**Scheme 79:**
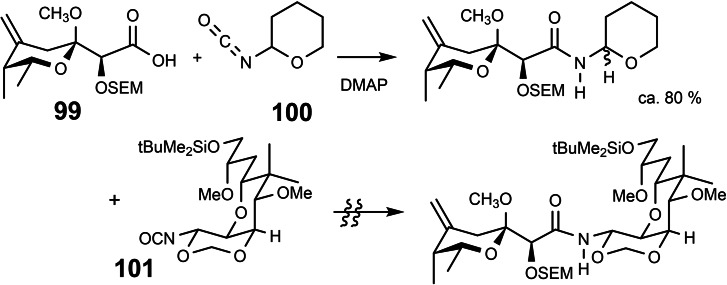
Attempted coupling of the left and right halves of mycalamide.

At this point we could finally test our envisaged (Scheme 73) coupling of protected pederic acid **99** with the right half of mycalamide B. The specially optimzed coupling between a carboxylic acid and an isocyanate^[109]^ proceeded smoothly between pederic acid **99** and the model isocyanate **100** ^[110]^ (Scheme 79). On reaction with the mycalamide building block **101** the reaction failed,^[106]^ probably because of steric overloading.

This answered the central question regarding a better coupling method for pederin and mycalamide syntheses. In a project, in which the critical experiment comes at the end of extended synthetic efforts, the probability of failure is maximized! This is, because with every successful step forward, the number of possible escape routes gets smaller. Hence, the project was terminated, also because I reached emeritus status and my labs were handed over to my successor.

**Scheme 80:**
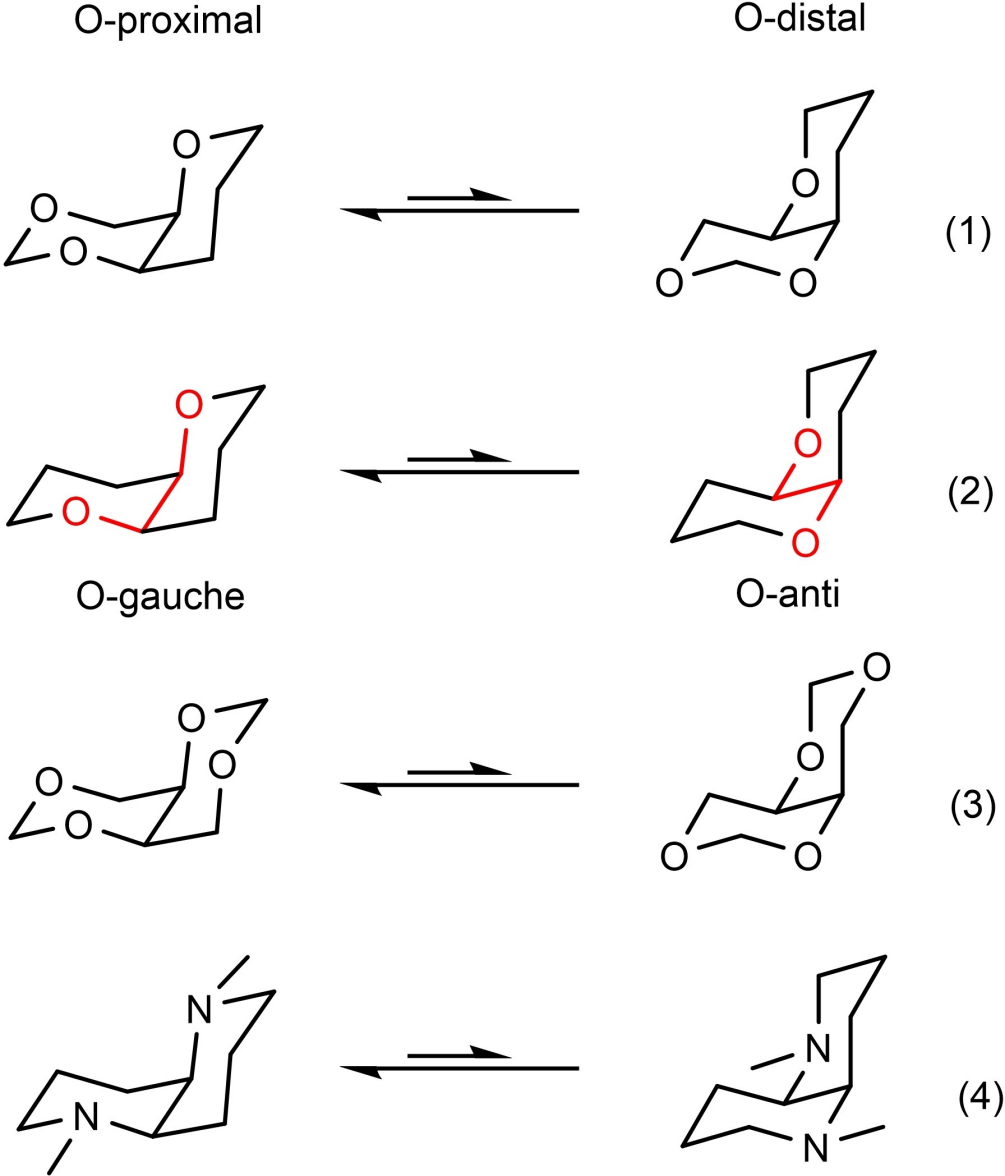
Conformer equilibria in heteroatom derivatives of cis‐decalin.

The project, though, had a sideline of hopefully more lasting impact. The success in the syntheses in the mycalamide manifold depended critically on the conformer equilibrium (1) in Scheme 80, specifically on shifting the equilibrium to either side by suitably placed substituents, cf. **94**/**95**. In order to learn the basics of such biconformational cis‐decalin equilibria, we studied substituent effects on the related equilibria (2)^[111]^ and (3).^[112]^ In the unsubstituted cases, the O‐proximal conformer is substantially favored due to the “gauche‐effect”. The gauche‐effect has simple steric (A‐values!) as well as stereoelectronic contributors. The latter are related to the anomeric effects in carbohydrate equilibria. The issue is, whether a C−H σ‐bond is a better or inferior hyperconjugative donor for a C−O σ*‐bond compared to a C−C σ‐bond. The former was found to be true on the basis of a high level computational study,^[113]^ and gives a handle to rationalize the position of such conformer equilibria. (Scheme 80)

These studies were then extended to the diaza‐cis‐decalin systems in equilibrium (4), interesting chiral analogs of the TMEDA additive in organolithium studies.^[114]^

## 12 Conformation Design

**Scheme 81:**
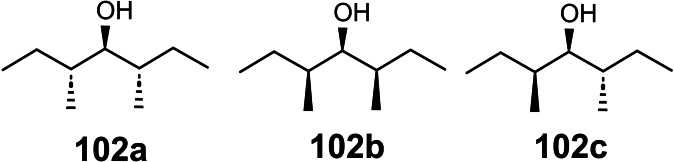
Diastereomeric stereotriads.

Studying stereoselective allylboration reactions of α‐chiral aldehydes, we run into mixtures of stereoisomeric products, such as the stereotriads **102**.^[115]^ The isomers displayed frequently eye‐catching differences in their nmr‐spectra. It was Jim Whitesell, in 1984 a visitor in our group, who suggested that the differences in the ^13^C‐nmr spectra could be a signature of different conformations, as the diastereomeric compounds **102** should adopt different *preferred conformations*.^[116]^ (Scheme 81)

Conformational analysis was then extended to compounds of the type **103** and allowed on comparison of the ^13^C‐nmr spectra of two stereoisomers an assignment of their relative configuration.^[117]^

**Scheme 82:**
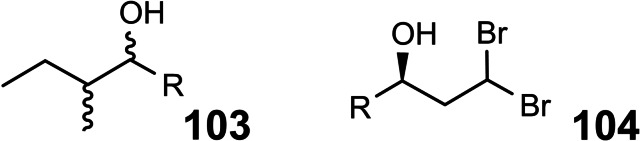
Compounds with preferred backbone conformations.

Studying the selective exchange of diastereotopic bromine atoms in 1,1‐dibromalkanes involved compounds of the type **104**. The compounds showed signatures in their nmr‐spectra, which clearly indicated the presence of *preferred conformations*.^[118]^ (Scheme 82)

**Scheme 83:**
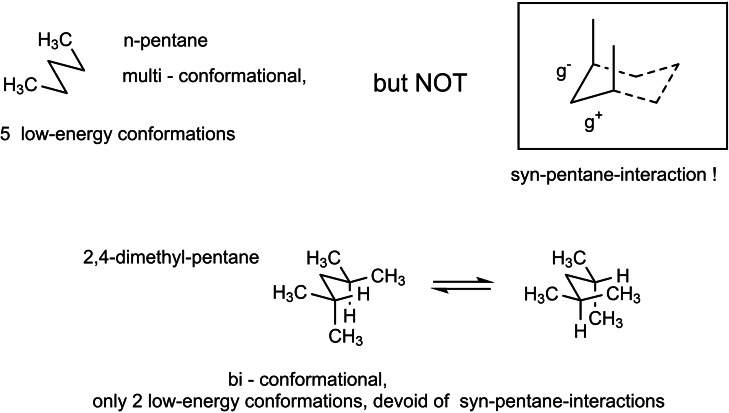
Pentane conformations.

We soon understood for compounds of the type **102**, resp. **104** the origin of the tendency to populate certain conformations in preference, the avoidance of syn‐pentane interactions.[^119^, ^120^] Note, that these compounds populate a multitude of conformations, but the preferred conformation to a considerably higher extent. (Scheme 83)

**Scheme 84:**
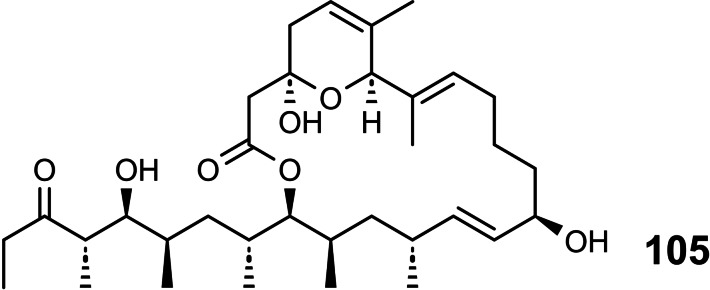
Venturicidine aglycon.

When occupied with the synthesis of polyketide natural products, we were amazed by the variety of patterns regarding the methyl branches. We wondered, what advantage in evolution the transition from acetate derived natural products to their propionate counterparts (those with methyl branches) may have caused. We surmised, that the local 2,4‐dimethylpentane‐subunits, of e. g. **105**, would by themselves and in combination with one another lead to preferred conformations of the molecular backbone, which could be beneficial for whatever biological activity. (Scheme 84)

**Figure 7:**
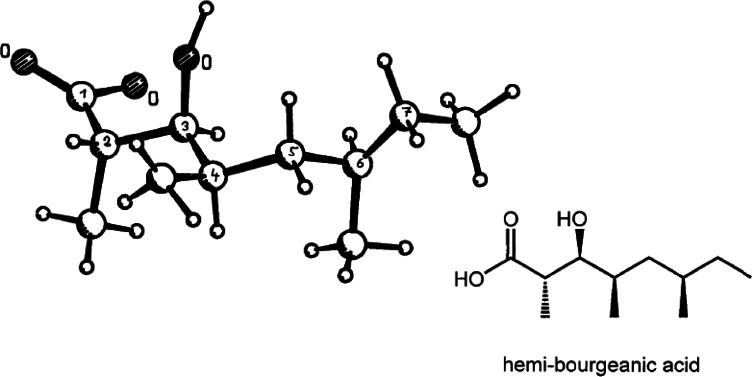
Structure of hemi‐bourgeanic acid, (Reproduced from Angew.Chem.**1992**, 104, 1147 with permission from John Wiley and Sons).

In due course we checked the (limited number of) crystal structure analyses of polypropionate natural products and found,^[121]^ that, with very few exceptions, the backbone conformation conformed to the one in which syn‐pentane‐interactions were avoided, cf. the structure of hemi‐bourgeanic acid:^[122]^ (Figure 7)

We had thus gained some rudimentary understanding of the conformation design of nature, which thereby attains a distinct folding of a fully flexible molecular backbone. These considerations kept us busy for several years. And even when the term „nature's conformation design“ was common in our discussions, I am ashamed that we realized only as late as 1992 that this knowledge asked for action. It was the slogan

Conformation Design

Nature does it !

And we ??

to initiate a research program. It coincided with the setting of a benchmark by our short synthesis of erythronolide. This led to increased demand by graduate students for projects in stereoselective synthesis. Yet by this time the attractive target molecules had become so complex, that they could not meaningfully be tackled by PhD‐theses which were limited to three years. It was out of question to extend PhD‐theses to 5–6 years as in the United States. Hence, we opted for a change from stereoselective synthesis of natural products to one of non‐natural products. The targets were „flexible molecules with defined shape“ originating from man‐made conformation design.

**Scheme 85:**
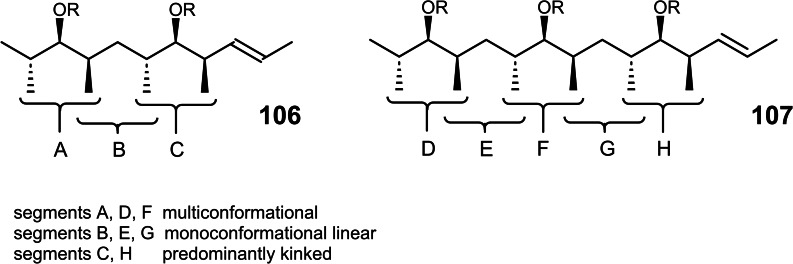
Conformational properties of models for polyketide natural products.

Right at the start, I published a review^[121]^ on conformation design, hoping for some feedback regarding to the contexts in which conformation design might be of value. There was no echo! So, we started anyhow. Fascinated by the crystal structure^[123]^ of venturicidine‐aglycon **105**, we were convinced that the substitution pattern **106** would fix a flexible alkane chain in an extended conformation. Yet after the elaborate synthesis of **106** and of the homologous compounds **107**, we were struck by the finding that conformational analysis revealed consistently a mixed situation:^[124]^ (Scheme 85)

Initially we asked why certain segments were multiconformational. Yet we found the answer only after reversing the question to why segments B, E, G were monoconformational. It is due to a conformation‐induction originating from the neighboring segment. The nature of this conformation‐induction was subsequently clarified in all detail.^[125]^ We thus still had to meet our goal of a flexible molecule with an *overall* extended conformation.

**Scheme 86:**
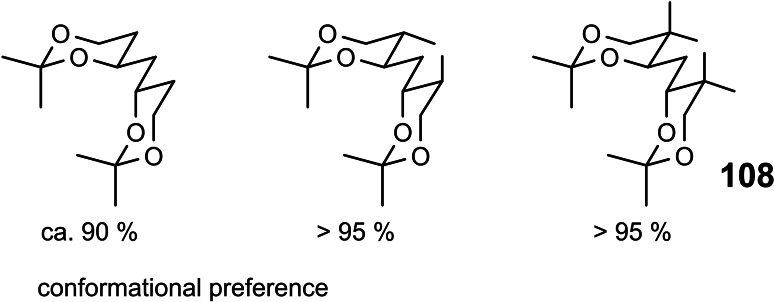
Building blocks with high conformational preferences.

In order to limit synthesis efforts, we considered to combine small conformationally preorganized molecules with one another. We chose as modules bis‐acetonides. Here, methyl substitution increased the conformational preference at the interring‐bond to >95 % on account of destabilizing syn‐hexane interactions in the other conformations.[^126^, ^127^] (Scheme 86)

**Scheme 87:**
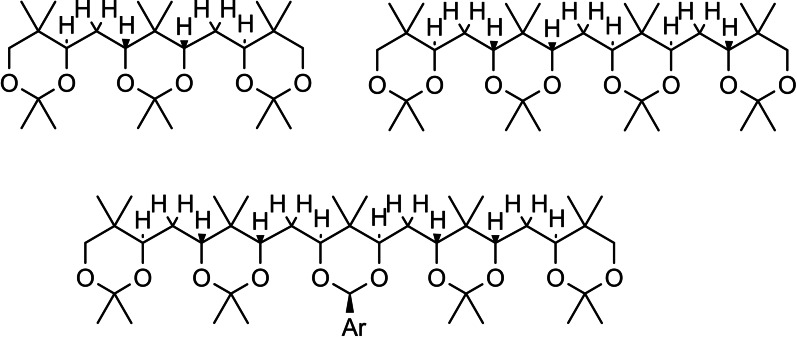
Poly‐1,3‐dioxanes with preference for fully extended conformations.

Building blocks with high conformational preferences such as compound **108** appeared as suitable moieties to be connected to longer chain artifacts with an overall preferred conformation. The compounds attained displayed indeed fully extended preferred conformations.^[128]^ (Scheme 87) However, the preference decreased with the number of interring bonds to be controlled. This pointed to the principal limit of this approach to conformation control: A preferred conformation entails loss of entropy, and this loss increases with the number of bonds to be controlled. This loss is counterbalanced by a penalty in enthalpy, which goes with the population of a *single* higher energy conformation. In the compounds shown this enthalpy penalty is approximately constant.

**Scheme 88:**
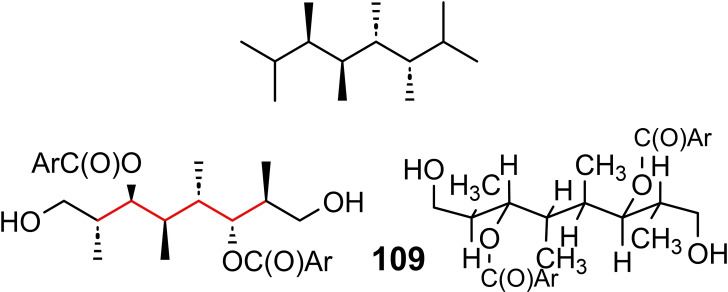
Compounds with fully extended conformation based on a hexamethyl‐octane backbone.

All compounds mentioned so far are based on the 2,4‐dimethylpentane backbone, in which rotation to an disfavored backbone conformation has the price tag of *one* syn‐pentane interaction. In a hexamethyl‐octane backbone the penalty is raised to *two* syn‐pentane interactions. (Scheme 88)

This move is effective, as compound **109** shows. It populates to >95 % the fully extended backbone conformation, despite being a completely open‐chain molecule.^[128]^

**Scheme 89:**
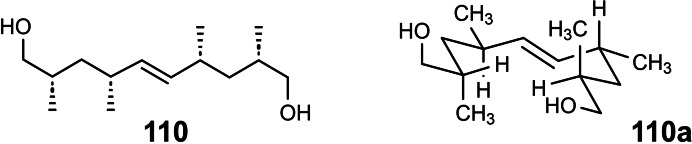
Compounds with a U‐shaped backbone conformation.

During our adventures in conformation control, we had also encountered substituent patterns that induce a kink in the molecular backbone. We hence sought to generate molecules with a preferred conformation favoring a kink. Compound **110** was accordingly expected to populate the U‐shaped conformation **110 a** with high preference. (Scheme 89)

**Scheme 90:**
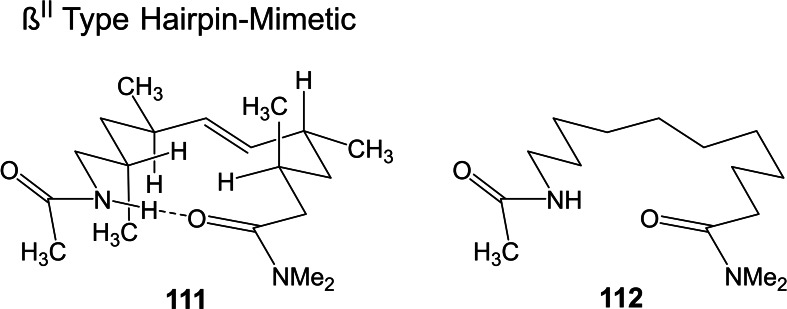
Open‐chain molecules as ß^II^ Type Hairpin mimetic.

There remained only a little step to take in order to reach a flexible ß^II^‐type hairpin mimetic **111**. The open chain compound **111** populates exclusively the H‐bonded conformation, whereas its counterpart **112**, devoid of any conformation controlling substituents, is multiconformational like freshly cooked spaghetti.^[129]^ (Scheme 90)

**Scheme 91:**
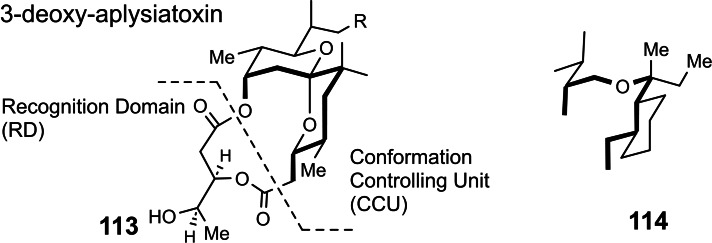
Design of a conformationally flexible backbone for a 3‐deoxy‐aplysiatoxin analogue.

We thus had learned to give a flexible open‐chain molecule a defined extended or kinked preferred backbone‐conformation by deliberate placement of substituents or by ring‐annellation. There remained the challenge to give an open chain molecule an arbitrarily chosen distinct preferred conformation. We were curious, how far one could progress with the above concepts in this direction. For a test, we chose as model 3‐deoxy‐aplysia‐toxin **113**. In this compound the dihydroxy‐pentanoyl unit is responsible for binding of **113** to its receptor. The remainder of the molecule simply has to present the dihydroxy‐pentanoyl unit correctly in space. We wanted to replace this rigid conformation‐controlling unit, by a flexible analogue that could display the dihydroxy‐pentanoyl unit correctly in space on account of a designed preferred conformation. (Scheme 91)

We documented the individual steps of such a design process in detail.^[130]^ It led us to the backbone **114**, which from several candidates appeared to best be able to replace the rigid conformation‐controlling unit in **113** as flexible counterpart. Initial experiments with model compounds showed, where further adjustments were needed.^[131]^ At that stage however, our research group was discontinued.

We had studied conformation design vigorously over more than ten years. The response of the scientific community was only meager. At best, we had been with these efforts ahead of time, in the worst case, this has led us into a dead end.

## 13 Combinatorial Catalysis

**Figure 8:**
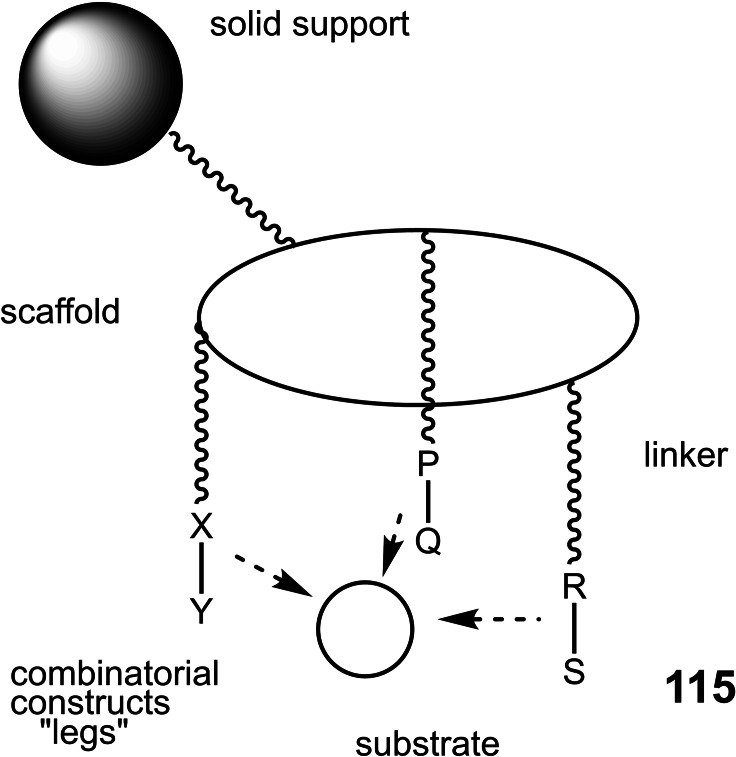
Envisioned scheme for a combinatorial approach to a molecular catalyst.

In 1995, Cesare Gennari in Italy and Tony Davis in Ireland, whom I knew from previous interactions, informed me that they planned together with others to apply for a European research network on „Combinatorial Approaches to Molecular Catalysts“. They inquired whether my group would like to participate as the network member from Germany. The research concept can be illustrated with reference to the following structure: (Figure 8)

The core is a molecular platform, to which „legs“ with functional groups at their ends are attached. These functional groups should cooperate in catalyzing a reaction, similar to the active center of an enzyme. The ensemble should be optimized by combinatorial variation of different „legs“.

At a preparatory meeting of the network partners it transpired that none of the groups made a claim for the linker moieties. This then fell – not unwelcome – to our Marburg team. As the linkers could be rigid, ordered flexible, or completely unordered, this opened an opportunity to bring in our experience in conformation design of open chain backbones.

The network was funded for over six years. Initially, our syntheses of conformationally preorganized linker building blocks^[132]^ were essentially service contributions for the network. At some time we began to wonder what consequence the presence or absence of conformational preorganization would have for the efficiency of a potential catalyst **115**? On catalysis, the „substrate“ of the ensemble **115** is the transition state of the reaction to be catalyzed. A model for the binding of a transition state could be the binding of an ordinary guest molecule. Hence, we studied to which extent the binding of a guest molecule depends on the conformational preorganization of the host molecule.

**Scheme 92:**
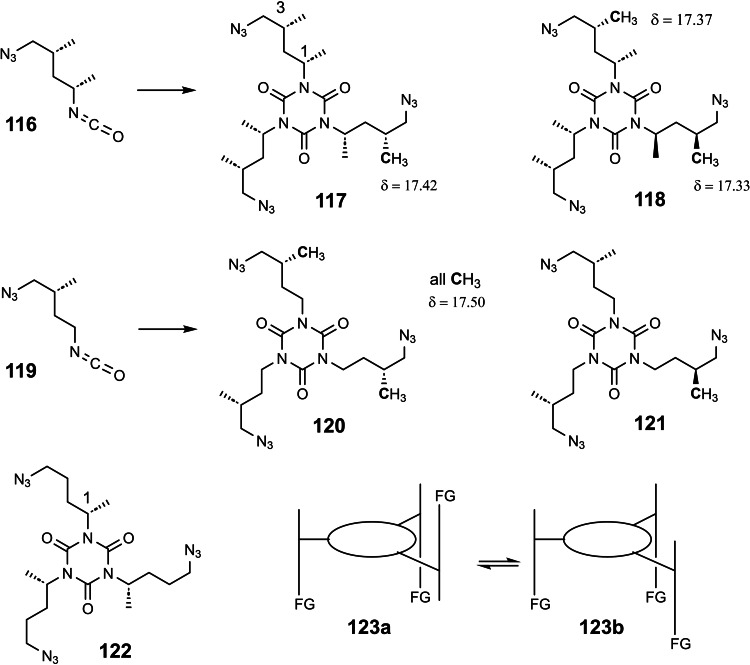
Triarmed platforms with legs differing in conformational preorganization.

We began with building our own platform. With respect to later combinatorial experiments, we chose to trimerize isocyanates derived from the linker moiety. Trimerization of enantiomerically pure **116** led to the uniform product **117**. Trimerization of racemic **116** yielded expectedly a mixture of compounds **117** and **118**. In similar manner, the isocyanate **119** was trimerized to compounds **120** and **121** respectively. Finally, we accessed as well the isocyanurate **122**.^[133]^ (Scheme 92)

The design was the following: In compound **120** (or **121**) the side arms of the isocyanurate can adopt all accessible conformations in space. In compound **122**, branching of the side arms in the 1‐position, causes the side arms to orient orthogonal to the platform as sketched in **123**, in order to avoid destabilizing 1,3‐allylic strain with the carbonyl groups of the platform. This holds as well for compound(s) **117** (and **118**), in which the second methyl branch additionally forces the side arms to adopt an extended conformation.

By serendipity we saw a confirmation for this design: the mixture of compounds **120** and **121** showed just a simple ^13^C‐nmr‐spectrum with a *single* methyl signal at δ=17,5 ppm. There was no reason to expect that a carbon atom would notice the change of relative configuration distant by nine skeletal bonds. The more we were struck by the nmr‐spectrum of compound **118**, whose 3‘‐methyl signals differed from one another and from those of compound **117**. This would be possible, when the methyl groups in the different side arms happen to be so close in space that they may communicate with one another. That observation demonstrates that the design is effective and that the preferred conformations of **117** and **118** corresponds to the intended arrangement **123**.

**Scheme 93:**
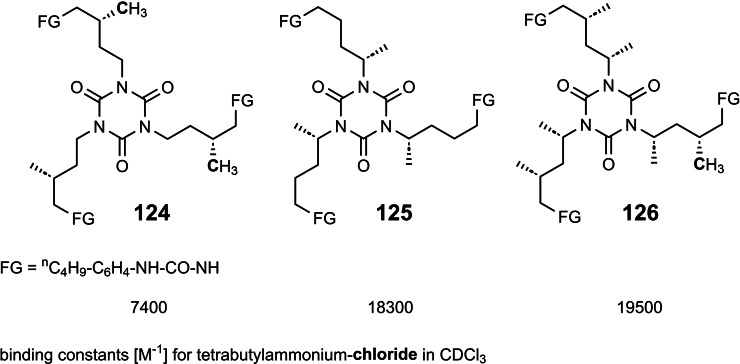
Chloride‐binding of triarmed hosts with different sidearm conformational preorganization.

Compounds **120**, **122**, and **117** were converted in to the host molecules **124**, **125**, and **126**, with urea sticking groups. The compounds were able to bind chloride ions, displaying the following binding constants: (Scheme 93)

The largest increase in binding energy resulted on change from a host with unordered side arms (**124**) to host **125**, in which the side arms are held orthogonal to the isocyanurate‐platform. Conformational preorganization within the side arms (vic. **126**) led to an additional, yet smaller increase. Likewise, the selectivity of the hosts towards different anions (chloride, bromide, and nitrate) increased in the same order.^[133]^

**Figure 9:**
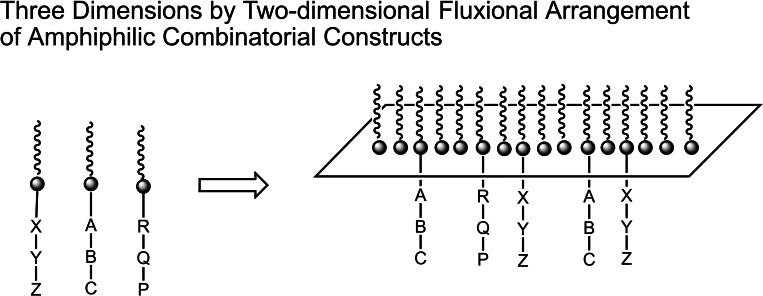
Model of dynamic combinatorial catalysis at an air/water interface.

The cooperative efforts in the research network led to first resuslts.^[134]^ In order to reach potential catalysts, the efforts followed the classical approaches of combinatorial chemistry. It was the time when J.‐M. Lehn published his fundamental concept of dynamic combinatorial chemistry.^[135]^ Fascinated by this concept, I wondered whether one could attain catalytically active molecular aggregates by dynamic combinatorial chemistry. The problem was, that the individual catalyst constituents have to move freely and independent of one another in order to meet the criteria of dynamic chemistry. However the constituents have to maintain a mutual orientation in space in order to generate enzyme like catalytically active centers. It occurred to me that amphiphiles in a Langmuir‐Blodgett film are mobile in two dimensions but ordered perpendicular with regard to of the surface. Could this be used to develop a system for dynamic combinatorial catalysts? (Figure 9)

**Figure 10:**
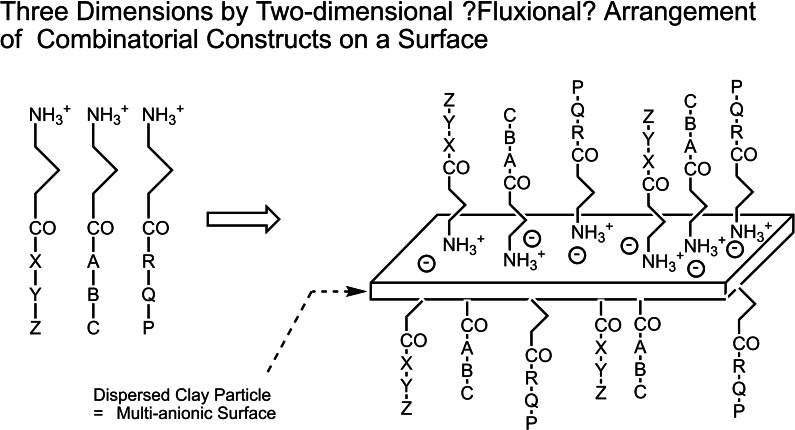
Model of dynamic combinatorial catalysis at a clay/water interface.

It took us some time to synthesize a set of amphiphilic components with functionalized end‐groups. We set out to test the concept on the aqueous saponification of diesters of phosphoric acid in a Langmuir‐trough. At this point a capital mistake in planning became obvious: The number of molecules of potential catalysts in a monolayer of ca. 20 cm^2^ is vanishingly (too) small compared to that of the substrate in 3.3 ml bulk solution. Even when the catalyst would provide an exorbitant k_cat_‐value, the attendant conversion of the diester saponification could not be differentiated from the uncatalyzed reaction or would remain below any detection limit. The ratio of active surface to volume of substrate solution was definitely too small with the approach of a Langmuir‐Blodgett film. One would have to increase the active surface by a huge factor! At this point I remembered my experience from my first postdoctoral studies, clay‐minerals. One could cover the surface of highly dispersed montmorillonite with catalyst components, whereby almost stoichiometric relations to a substrate solution could be reached,^[136]^ i. e. a change to the following system: (Figure 10)

Such a change of the system would, however, require synthesis of a new set of catalyst components, ones carrying cationic sticking groups. Of even greater concern was that we had no information on the lateral mobility of cationic adsorbates on a negatively charged montmorillonite surface – a prerequisite for dynamic combinatorics. Therefore, we stopped our activities at this point, which coincided with the end of funding of the European research network, and with the handover of our labs to my successor – all in time, before I could make any further consequential planning mistakes.

## 14 Conclusion

**Figure 11:**
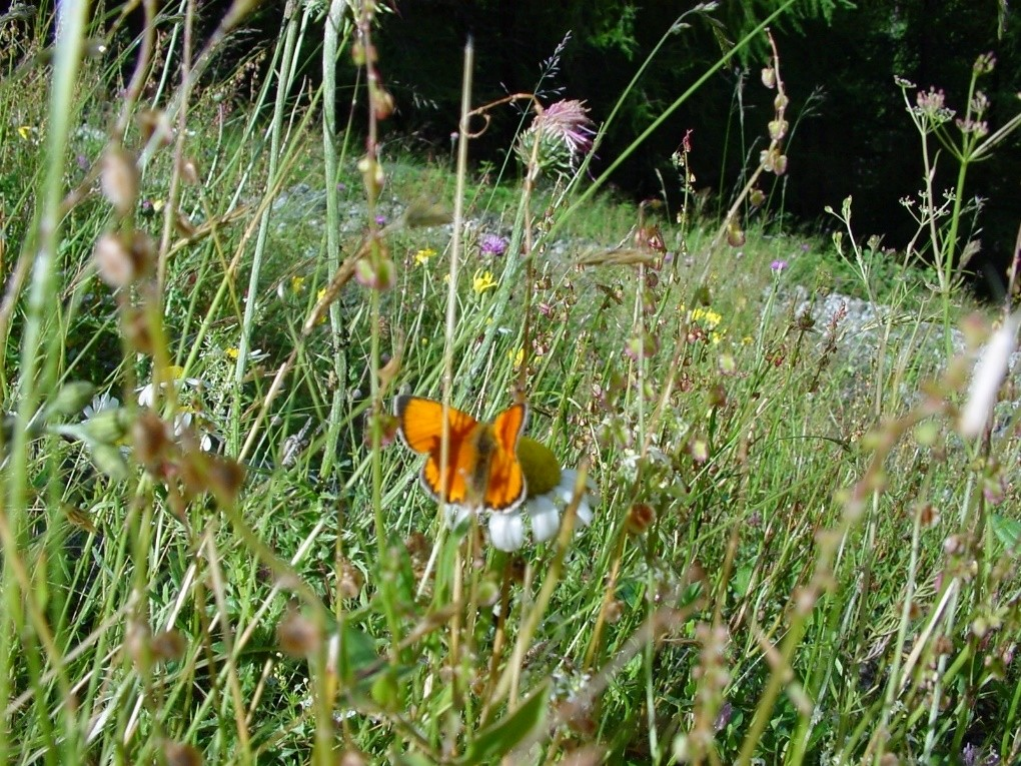
In search of nectar.

Reflection on the previous twelve sections leaves no doubt that there is not any systematic approach or any golden thread. That means that the initiation of the various research projects in our group was much like a butterfly on a blooming meadow (Figure 11), looking for the freshly opened blossom full of nectar, a blossom not having been visited by many other butterflies before. The reflection shows that the conception of the research projects was triggered by diverse factors, such as an unusual experimental result, unsolved problems encountered in the preparation of teaching materials, current developments in Organic Chemistry, or personal interactions in the scientific community. Collaboration with other groups became more prevalent over time. It is perhaps a privilege of my generation that funding considerations were mostly marginal.

The conception of a project marked only an entry point. Frequently the initial goals were getting out of sight, when unexpected insights shifted the objectives of the project. In this kind of curiosity‐driven research, projects – except for the last ones – were not ended intentionally; they just dried up when the interest shifted to another project in an exciting exploratory phase. This was much more fascinating than continuing a project through an extended harvesting phase.

While my experiment‐based research projects ended in 2001 with the break‐up of our research group, this did not necessarily hold for my research activities; I had to fall back to the second line, that organic chemistry is not only an experimental science, but also one with which to deal intellectually. As the focus of my activities had been for over forty years in the realm of organic synthesis, it suggested itself to consider organic synthesis in depth, e. g. the art of synthesis planning. My existent teaching materials could readily be converted into a text:

R. W. Hoffmann, *Elemente der Syntheseplanung*, 1st ed., Elsevier, München, **2006**, pp. 1–202.

R. W. Hoffmann, *Elements of Synthesis Planning*, Springer, Berlin‐Heidelberg, 2009.

The approach to the synthesis of complex target structures is a historical process:

R. W. Hoffmann, Natural Product Synthesis: Changes over Time, *Angew. Chem*. **2013**, *125*, 133–140; *Angew. Chem. Int. Ed*. **2013**, *52*, 123–130.

R. W. Hoffmann, Complex Molecule Synthesis, a Personal View, *Isr. J. Chem*. **2018**, *58*, 73–79.

Tracing the historical changes in the synthesis of complex target structures revealed where improvements became mandatory:

R. W. Hoffmann, Protecting‐Group‐Free Synthesis, *Synthesis* 2006, 3531–3541.

N. Z. Burns; P. S. Baran; R. W. Hoffmann, Redox Economy in Organic Synthesis, *Angew. Chem*. **2009**, *121*, 2896–2910; *Angew. Chem*., *Int. Ed*. **2009**, *48*, 2854–2867.

T. Newhouse; P. S. Baran; R. W. Hoffmann, The Economies of Synthesis, *Chem.Soc.Rev*. **2009**, *38*, 3010–3021.

R. W. Hoffmann, Synthesis Without Protecting Groups, in *Handbook of Green Chemistry*, *Green Processes* (Eds.: P. T. Anastas; C.‐J. Li), Wiley‐VCH, Weinheim, **2012**, pp. 215–235.

R. W. Hoffmann, Streamlining Organic Synthesis for the 21st Century, *Zhurnal Organicheskoi Khimii* **2012**, *48*, 631–642; *Russian Journal of Organic Chemistry* **2012**, *48*, 625–637.

R. W. Hoffmann, Biomimicry in Organic Synthesis, in *Bioinspiration and Biomimicry in Chemistry; Reverse‐Engeneering Nature* (Ed.: G. F. Swiegers), J.Wiley & Sons, New York, **2012**, pp. 419–453.

Moreover, distinct aspects of Organic Synthesis merited attention:

R. W. Hoffmann, meso‐Compounds: Stepchildren or Favorite Children of Stereoselective Synthesis, *Angew. Chem*. **2003**, *115*, 1128–1142; *Angew. Chem*., *Int. Ed*. **2003**, *42*, 1096–1109.

R. W. Hoffmann, Stereoselective Synthesis Using Diastereotopic Groups, *Synthesis* 2004, 2075–2090.

R. W. Hoffmann, General Principles of Diastereoselective Reactions: Acyclic Control of Diastereoselectivity, in *Comprehensive Chirality* (Eds.: E. M. Carreira; H. Yamamoto), Elsevier, Amsterdam, **2012**, pp. 65–96.

R. W. Hoffmann, Markovnikov Free Radical Addition Reactions, a sleeping beauty kissed to life, *Chem.Soc. Rev*. **2016**, *45*, 577–583.

P. R. Blakemore; R. W. Hoffmann, Formation of Olefins by Eliminative Dimerization and Eliminative Cross‐Coupling of Carbenoids: A Stereochemical Exercise, *Angew. Chem*. **2018**, *130*, 396–413; *Angew. Chem. Int. Ed*. **2018**, *57*, 390–407.

N. A. Doering, R. Sarpong, R. W. Hoffmann, A Case for Bond‐Network Analysis in the Synthesis of Bridged Polycyclic Complex Molecules: Hetidine and Hetisine Diterpenoids, *Angew. Chem*. **2020**, *132*, 10810–10820; *Angew. Chem. Int. Ed*. **2020**, *59*, 10722–10731.

In dealing with these topics in the years following getting emeritus status in 2001, I learned with gratitude how rewarding a purely intellectual interaction with Organic Synthesis might be. Likewise, I learned that a look back to history lets one recognize more clearly, where we stand today:

R. W. Hoffmann, Classical Methods in Structure Elucidation of Natural Products, Wiley‐VHCA, Zürich, **2018**, pp 1–265.

What made me sustain an almost life‐long occupation with research in Organic Chemistry? The ability to find repeatedly new challenges? The unique joyful rewards of having a Eureka moment, the joy of reaching a difficult benchmark? Yes, but as most researchers will agree, such rewards come only very rarely, too seldom, to base a scientists life activity on. In contrast, joyful rewards are much easier to attain in education in general, when teaching is not “to merely present facts”, but rather to outline how combination of facts may allow solving problems, and how to recognize significant problems. Moreover, as implied above, teaching may provide valuable inspirations and starting points for research.

## 15 Coda

When looking back at my scientific career, I realize that I never stood before closed doors. Who (singular) or who (plural) have been my promoters, I do not know. They provided just the opportunity for me to go through that door or to walk past it. I am very grateful to them, for rendering my career so smooth. I realized, that a way to express my gratitude, would be to act as promoter for the scientists of the next generation.

It thus became almost a compulsion for me to try to spot young chemists with outstanding talent in the German chemistry scene. The earlier, i. e. at the PhD or podstdoc level, the better. I would monitor their development, invite them for a seminar to Marburg, and try to foster their academic and scientific path. Whatever insignificant my influence may have been, it gave me deep joy to see them develop into full‐fledged and respected scientists. This is the aspect of my scientific achievements that I value the highest.
